# Host-Plant-Mediated Effects on the Demography, Parasitism Rates and Functional Response of *Bracon hebetor* on *Spodoptera frugiperda*

**DOI:** 10.3390/insects17101085

**Published:** 2026-10-20

**Authors:** Gouranga Saw, B. V. Jayanth, Shashikala Mahadev, Gundreddy Rajareddy, Edula Udaykumar, Bishwajeet Paul, Mallikarjuna Jeer, Rashid Mumtaz Khan, Dhirendra Kumar Gupta

**Affiliations:** 1 Biological Control Laboratory, Division of Entomology, Indian Council of Agricultural Research- Indian Agricultural Research Institute, New Delhi 110012, India; gourangasaw8612@gmail.com (G.S.); bvjayanth555@gmail.com (B.V.J.); shashikalam230699@gmail.com (S.M.); rajareddygundreddy422@gmail.com (G.R.); udayernesto@gmail.com (E.U.); 2 Central Muga Eri Research and Training Institute, Lahdoigarh, Jorhat 785700, India; 3 School of Crop Health Biology Research, ICAR-National Institute of Biotic Stress Management, Raipur 493225, India; mallikarjuna.j@icar.org.in; 4 Department of Chemistry, College of Science, Qassim University, Buraydah 51452, Saudi Arabia; 5 Department of Statistics and Operations Research, College of Science, Qassim University, Buraidah 52571, Saudi Arabia; d.gupita@qu.edu.sa

**Keywords:** biological control, host plant effects, tritrophic interactions, demographic analysis, parasitism rates, integrated pest management

## Abstract

Tritrophic interactions among host plants, herbivores and parasitoids can influence biological control efficacy, yet plant-mediated effects on parasitoid performance against the invasive fall armyworm, *Spodoptera frugiperda*, are poorly understood. We evaluated the effects of maize and castor on the life-history traits, demographic parameters, parasitism, functional response and population projection of the larval ectoparasitoid *Bracon hebetor*. Laboratory and greenhouse experiments used *S. frugiperda* larvae reared on the two host plants. Age-stage, two-sex life tables, parasitism indices, population projections and functional response models were used to compare parasitoid performance. Parasitoids developing on maize-fed larvae had significantly shorter developmental periods, higher fecundity (247.09 ± 0.41 eggs), higher intrinsic (*r* = 0.217 ± 0.008 day^−1^) and finite (λ = 1.242 ± 0.011) rates of increase and a shorter generation time (*T* = 20.26 ± 0.289 days) than those on castor-fed larvae, whereas the net reproductive rate (*R*_0_ = 81.54 ± 11.55) and gross reproductive rate did not differ significantly. Effective parasitism and killing rates were higher on maize-fed larvae in both laboratory and greenhouse assays; non-effective parasitism did not differ significantly. Type II functional responses were observed on both host plants, and searching efficiency tended to be higher and handling time shorter on maize-fed larvae (Holling’s disc equation: *a* = 1.125 vs. 0.787; *T*_h_ = 0.0556 vs. 0.0817 days). Projections suggested faster parasitoid population growth and greater cumulative host killing on maize-fed larvae. These findings suggest that host-plant-mediated effects should be considered when optimizing sustainable biological control of *S. frugiperda*.

## Simple Summary

Host plant–herbivore–parasitoid interactions can influence the effectiveness of biological control against insect pests. The invasive fall armyworm, *Spodoptera frugiperda*, develops on diverse host plants, which may alter its suitability as a host for natural enemies. In this study, we examined the effects of maize and castor on the development, reproduction, population growth, parasitism, host killing and functional response of the larval ectoparasitoid *Bracon hebetor* developing on fall armyworm larvae. Under laboratory conditions, parasitoids developing on maize-fed larvae showed shorter developmental periods, longer adult longevity, greater fecundity and higher intrinsic and finite rates of increase than those developing on castor-fed larvae. Effective parasitism and killing rates were also higher on maize-fed larvae in laboratory and greenhouse assays, whereas non-effective parasitism did not differ significantly. The parasitoid showed a type II functional response on both host plants, and searching efficiency tended to be higher and handling time shorter on maize-fed larvae. Population projections suggested faster parasitoid population growth and greater cumulative host killing on maize-fed larvae. These findings indicate that the host plant on which fall armyworm develops can substantially influence parasitoid performance and biological control potential, providing important insights for optimizing *B. hebetor*-based management of fall armyworm.

## 1. Introduction

Fall armyworm (FAW; *Spodoptera frugiperda* (J.E. Smith); Lepidoptera: Noctuidae) is a highly polyphagous lepidopteran pest originally confined to the subtropical and tropical Americas and has become a significant invasive pest worldwide. Following its first detection in West Africa in 2016, the pest has expanded at an alarming rate, establishing populations in over 44 African countries [1,2]. By 2018–2019, it had further invaded into Asia, including India, Japan, Korea, and Australia [3]. Its strong migratory ability, exceptional reproductive output and extremely wide host range exceeding 350 plant species have allowed it to colonize and thrive in a broad array of agricultural habitats [4,5,6]. Among its hosts, maize (*Zea mays* L.) suffers the greatest damage, with annual losses estimated at 8.3–20.6 million tonnes [7], threatening global food security and rural livelihoods [8,9]. Its establishment in areas lacking natural enemies has triggered severe outbreaks [10], and intense feeding often leads to indiscriminate use of hazardous or banned pesticides [11]. Therefore, economically and environmentally sustainable management is essential for conserving biodiversity, safeguarding global food supplies, and promoting human well-being [12]. A variety of natural enemies (NEs) are associated with FAW [13], offering substantial potential for long-term, sustainable pest suppression [14].

*Bracon* is the most species-rich genus within Braconinae, comprising 32 species in India and over 800 globally [15,16]. Members of this genus display a wide range of host specificity, from strict specialists restricted to a single host to highly polyphagous species parasitizing over a hundred lepidopteran hosts across diverse ecosystems [17]. *Bracon hebetor* Say, 1836 (Hymenoptera: Braconidae) is among the most widespread species, occurring throughout Asia, Africa, Europe, Oceania and North America [18]. Females paralyze their hosts with venom and oviposit on or near the immobilized larvae during the wandering phase [19], after which the developing larvae feed externally and eventually kill the host [20]. Mature larvae move away from the cadaver and spin white cocoons for pupation [21]. This gregarious idiobiont ectoparasitoid attacks a broad range of lepidopteran pests, including *Tuta absoluta* (Meyrick, 1917) (Lepidoptera: Gelechiidae), *Heliocheilus albipunctella* (de Joannis, 1925) (Lepidoptera: Noctuidae), *Spodoptera litura* (Fabricius, 1775) (Lepidoptera: Noctuidae), and *Helicoverpa armigera* (Hübner, 1808) (Lepidoptera: Noctuidae), as well as key stored-product moths such as *Sitotroga cerealella* (Olivier, 1789) (Lepidoptera: Gelechiidae), *Ephestia kuehniella* Zeller, 1879 (Lepidoptera: Pyralidae), *Plodia interpunctella* (Hübner, 1813) (Lepidoptera: Pyralidae), and *Galleria mellonella* (Linnaeus, 1758) (Lepidoptera: Pyralidae) [22,23,24,25,26,27].

Tritrophic interactions among host plants, herbivores, and natural enemies (NEs) play a fundamental role in shaping ecological processes and the effectiveness of biological control [28]. Host plant quality can indirectly influence the survival, development, behaviour, and reproductive performance of parasitoids by altering the nutritional and physiological characteristics of their herbivore hosts [29,30,31,32]. Consequently, plant-mediated changes in host quality may affect parasitoid efficiency, host susceptibility, and ultimately the success of biological control programmes [33,34,35]. Understanding these bottom-up effects is therefore essential for accurately evaluating parasitoid performance and optimizing integrated pest management (IPM) strategies.

Behavioural responses, especially the functional response, have a significant impact on the efficiency of parasitoids in regulating host populations. Functional response describes the relationship between the number of hosts attacked or parasitized by an individual parasitoid and increasing host density over a given period, thereby reflecting its host-searching efficiency and capacity for density-dependent regulation. Consequently, it is widely used to assess the biological control potential of parasitoids [36]. Functional response parameters are influenced by various abiotic and biotic factors, including plant type, parasitoid age, host species, temperature, and laboratory conditions [37,38,39,40]. For instance, variation in the functional response of *H. hebetor* has been demonstrated across different instars of the rice moth, *Corcyra cephalonica* (Stainton) (Lepidoptera: Pyralidae) [41]. Understanding these responses is critical, as simulation studies indicate that the optimal release strategy of parasitoids depends on their functional response type [42]. In addition to behavioural responses, demographic analyses based on the age-stage, two-sex life table provide a comprehensive framework for evaluating parasitoid performance by integrating survival, development, fecundity, longevity, and population growth. Unlike conventional life tables, this approach accounts for stage differentiation and both sexes, enabling a more realistic assessment of parasitoid population dynamics and biological control potential. Therefore, integrating functional response with life table analysis provides a comprehensive evaluation of parasitoid efficacy under different host-plant-mediated trophic conditions.

Despite the recognized influence of host type on parasitoid performance, no study has evaluated how host-plant-mediated trophic interactions simultaneously influence the behavioural responses (functional response), life table parameters, and parasitism potential of *B. hebetor* when reared on *S. frugiperda*. Consequently, the plant-mediated mechanisms governing the demographic performance and host-searching behaviour of *B. hebetor* remain largely unexplored. Maize is a highly suitable host plant for FAW, whereas castor (*Ricinus communis* L.) contains defensive allelochemicals, such as ricin and ricinine, that can impair herbivore growth and may reduce host quality for natural enemies [43,44]. We therefore hypothesized that FAW larvae reared on maize would be a more suitable host for *B. hebetor* than those reared on castor, resulting in shorter development, higher survival and reproduction, greater parasitism and killing rates, and a more efficient functional response (higher searching efficiency and shorter handling time). The corresponding null hypothesis was that host plant identity has no effect on these traits. To test this, the specific objectives were to: (i) compare the development, survival, longevity and fecundity of *B. hebetor* on maize- and castor-fed FAW larvae; (ii) estimate age-stage, two-sex life table parameters; (iii) quantify effective parasitism, non-effective parasitism and killing rates under laboratory and greenhouse conditions; (iv) determine the type and parameters of the functional response; and (v) project parasitoid population growth and cumulative host killing over 60 days. The findings are expected to support the effective deployment of *B. hebetor* in biological control programmes against the globally invasive pest *S. frugiperda*.

## 2. Materials and Methods

### 2.1. Raising of Host Plants

Host plants of maize (cv. PJHM-1) and castor (cv. GCH-8) were established under open-field conditions at the Division of Entomology, ICAR–Indian Agricultural Research Institute (ICAR-IARI), New Delhi, India, during the 2024 cropping season. The crops were raised following the recommended agronomic practices for their respective cultivars, including timely irrigation, fertilization, and manual weed management, without the application of any plant protection measures. Fresh, healthy, untreated leaves were harvested from maize plants from 21 days after sowing (DAS) onwards and from castor plants from 40 DAS onwards. Leaves were collected daily and supplied fresh for rearing *Spodoptera frugiperda* throughout the experimental period.

### 2.2. Rearing of Spodoptera frugiperda

*Spodoptera frugiperda* egg masses were gathered from the fields of maize at ICAR-IARI, New Delhi, and brought to the Biological Control Laboratory. Newly emerged larvae were maintained under controlled laboratory conditions [27 ± 2 °C, 65 ± 5% RH and photoperiod (12L:12D)] and provided daily with fresh leaves of either maize or castor, following the standard procedures [43]. From the 3rd instar onward, larvae were individually placed to plastic vials (60 mm × 45 mm) to prevent cannibalism and maintained until pupation. Pupae were placed into ventilated plastic jars (20 × 15 cm) secured with muslin cloth. After adult emergence, moths were provided with a 10% honey solution on cotton wicks as a carbohydrate source to support adult longevity and fecundity, and crumpled paper was placed inside the jars as an oviposition substrate [45]. The same adult diet and oviposition substrate were used for both host plant colonies so that adult feeding could not bias the comparison between treatments. Independent colonies were reared on each host plant for a minimum of three generations to achieve dietary acclimation before their use in experiments.

### 2.3. Rearing of Corcyra cephalonica

A laboratory culture of *Corcyra cephalonica* (Stainton, 1866) (Lepidoptera: Pyralidae) was maintained as a factitious host for the routine mass rearing and maintenance of *Bracon hebetor* colonies before initiating experiments with *Spodoptera frugiperda*. The culture was reared on crushed maize grains meant for human consumption following the established rearing protocols [46]. The crushed grains were heat-sterilized at 100 °C for 3 h to eliminate contaminants, after which dried yeast (2 g kg^−1^) was added to enhance nutritional quality. Rearing boxes were filled with 4.0 kg of processed grains and inoculated with approximately 10,000 eggs (0.5 cc) of *C. cephalonica*. The cultures were maintained at 27 ± 2 °C, 60 ± 5% RH. Every day, adults were gathered and placed into oviposition jars. Larvae from the rearing boxes were cleaned and used for subsequent rearing of the larval parasitoid, *B*. *hebetor*.

### 2.4. Rearing of Bracon hebetor

*Bracon hebetor* culture was established using pupal cards procured from Dr. YSR Horticultural University, Ambajipeta, Andhra Pradesh, India. The *B. hebetor* stock colony was maintained on 5th–6th-instar larvae of the rice moth, *Corcyra cephalonica*, a factitious host. Mass-rearing the parasitoid directly on *S. frugiperda* is impractical for a stock colony because FAW larvae are cannibalistic and must be reared individually, which is labor-intensive, and because maintaining FAW throughout the year requires continuous supplies of fresh maize and castor leaves. In contrast, *C. cephalonica* can be reared in bulk on stored grain, is available year-round, and supports efficient multiplication of *B. hebetor*. The parasitoids were reared using the sandwich technique [47]. Adults were introduced into glass jars (15 × 10 cm) covered with muslin cloth, and twenty 5th–6th-instar *C. cephalonica* larvae were placed on the muslin surface and covered with an additional layer. Adult parasitoids were provided with a 20% honey solution on cotton swabs [48]. After 24 h of exposure, parasitized larvae were transferred to Petri dishes lined with filter paper for pupation, and the resulting pupae were transferred to fresh jars until adult emergence. To eliminate potential host-conditioning and maternal effects associated with the factitious host, newly emerged *B. hebetor* adults were subsequently transferred to *S. frugiperda* larvae reared on maize and castor, and the colonies were maintained for three successive generations on each host plant system before initiating the experiments.

### 2.5. Life Table Parameters

To study the life table traits of *B. hebetor*, newly emerged females (≤24 h) obtained from FAW larva reared on maize or castor were individually paired with males from the same host plant systems. Fifth-instar FAW larvae were selected because larger host instars generally provide greater nutritional resources and support improved parasitoid development and reproductive performance, although they may exhibit stronger immune defenses than younger instars. Previous studies have shown that the overall suitability of older instars for idiobiont ectoparasitoids reflects a balance between increased host resources and host immune responses [19,49]. For each host plant, two sets of fifty culture bottles (50 mL; 3.5 cm × 6 cm), each containing fresh leaves and a single larva, were grouped together inside an acrylic cage (1 m × 1 m × 0.5 m). Each group of bottles was covered with a 50 × 50 cm stainless-steel woven wire cloth (8-mesh) to restrict larval escape while allowing parasitoid access. Ten mated *B. hebetor* females were released into each cage along with a honey source to enable parasitization. After 24 h of exposure, each paralyzed host larva bearing externally laid *B. hebetor* eggs was individually transferred to a Petri dish lined with filter paper. Because *B. hebetor* is a gregarious ectoparasitoid that deposits multiple eggs externally on a single host, only one egg was retained on each larva, while the remaining eggs were carefully removed to establish a standardized cohort of 100 individuals per host plant treatment. Larval development, pupation, adult emergence, and mortality were recorded daily. Newly emerged adults were sexed, paired (one female × one male), and supplied daily with five fifth-instar FAW larvae from the corresponding host plant treatment. Observations were continued until the death of all individuals to record adult longevity, fecundity, and mortality.

### 2.6. Parasitism Potential

Parasitism potential of *B. hebetor* females was evaluated using the same experimental setup employed for life table assessments. Mated females from each host plant treatment were placed in plastic jars (15 cm × 10 cm) containing five FAW larvae, which were maintained separately as described in the life table study. Parasitoid females were allowed to oviposit for 24 h and were then transferred daily to fresh jars containing five new fifth-instar FAW larvae until their death. After each transfer, exposed larvae were placed individually in Petri dishes, where parasitized larvae bearing externally laid eggs (effective parasitism), paralyzed larvae without eggs (non-effective parasitism), and total paralyzed larvae (killing rate) were recorded daily. Effective parasitism, non-effective parasitism, and killing rates were evaluated using the CONSUME-MS Chart [50].

Further, to validate the parasitism potential under semi-field conditions, a greenhouse experiment was conducted on maize (~3 weeks old) and castor (~6 weeks old) plants grown in pots (15 × 10 cm) under natural, insecticide-free conditions. For each host plant, five plants were placed together in a single cage (90 × 60 cm), and five independent cages were used as replicates. Each cage was infested with 20 fifth-instar FAW larvae (four larvae per plant). After a 24 h feeding period, five 3-day-old mated *B. hebetor* females were released into each cage and allowed to parasitize for 24 h [49]. Thus, five independent replicate cages were maintained for each host plant, and % effective parasitism, non-effective parasitism, and killing rate were quantified based on the number of paralyzed larvae.

### 2.7. Functional Response

The functional response of *B. hebetor* was evaluated against FAW larvae (fifth-instar) reared on maize and castor using 3-day-old mated female wasps. Host densities of 2, 4, 8, 16, 32, and 64 were tested, with individual larvae placed on fresh leaves in 50 mL culture bottles and grouped within an acrylic cage as described above. Each host plant treatment was replicated five times. Prior to introducing the parasitoid, cotton wicks soaked in 20% honey solution were provided as a food source. A single female wasp was then released into each cage. After 24 h, the parasitoid was removed, and the number of parasitized larvae (effective + non-effective parasitism) was recorded.

### 2.8. Life Table Analysis

Life table data of *B. hebetor* across all developmental stages on the *S. frugiperda*–host plant complexes were analyzed using the age-stage, two-sex life table framework [51] implemented in the TWOSEX-MS Chart [52]. Based on the established methodology [51], we estimated age-specific survival rate (*l_x_*) and fecundity (*m_x_*), age-stage-specific survival rate (*S_xj_*) and fecundity (*f_xj_*). Key population growth metrices—including gross (*GRR*) and net (*R*_0_) reproductive rate, intrinsic (*r*) and finite (*λ*) rate of increase, doubling time (*DT*) and mean generation time (*T*)—were subsequently derived.

*S_xj_*, defined as the probability of a newly laid egg reaching age *x* and stage *j*, was computed as:

$$S_{\mathrm{xj}}=\frac{n_{\mathrm{xj}}}{n_{01}}$$

where *n*_01_ and *n_xj_* represent the initial cohort size and the number of individuals surviving to the specified age and stage, respectively.

*l_x_*, or the probability of a newborn egg surviving to age *x*, and *m_x_* were calculated as:

$$l_{x}=\sum_{j=1}^{\beta }S_{\mathrm{xj}};m_{x}=\frac{\sum_{j=1}^{\beta }S_{\mathrm{xj}}\;f_{\mathrm{xj}}}{\sum_{j=1}^{\beta }S_{\mathrm{xj}}}$$

where *β* is number of developmental stages and *f_xj_* is the daily fecundity of an individual at specified age and stage.

*R*_0_, defined as total number of offspring produced by an individual over its lifetime, was calculated as:

$$R_{0}=\sum_{x=0}^{\infty }l_{x}\;m_{x}=\sum_{x=0}^{\infty }\sum_{j=1}^{\beta }S_{\mathrm{xj}}\;f_{\mathrm{xj}}$$

The intrinsic rate of increase (*r*) was determined by applying an iterative bisection approach to the Euler–Lotka equation [53]:

$$r=\sum_{x=0}^{\infty }e^{-r(x+1)}l_{x}m_{x}=1$$

The finite rate of increase (*λ*), mean generation time (*T*), and doubling time (*DT*) were estimated using the following formulas:

$$\lambda =e^{r};T=\frac{\ln\left(R_{0}\right)}{r}\;\mathrm{and}\;\mathrm{DT}=\frac{\ln2}{r}$$

The age-stage-specific life expectancy at age *x* and stage *j* (*e_xj_*), representing the expected remaining lifespan of an individual, was calculated following [54] and is represented as:

$$e_{\mathrm{xj}}=\sum_{j=x}^{\infty }\sum_{y=j}^{\beta }{s^{\prime }}_{\mathrm{iy}}$$

where *s′_iy_* is the probability that an individual will survive to age *i* and stage *y*, assuming *S_xj_* = 1.

The age-stage-specific reproductive value (*v_xj_*), explained as the expected contribution of an individual of age *x* and stage *j* to the future population [55], was computed using following equation:

$$v_{\mathrm{xj}}=\frac{e^{r\left(x+1\right)}}{s_{\mathrm{xj}}}\sum_{i=x}^{\infty }e^{-r\left(i+1\right)}\sum_{y=j}^{\beta }{s^{\prime }}_{\mathrm{iy}}f_{\mathrm{iy}}$$

### 2.9. Effective Parasitism Rate (EPR) Analysis

EPR was defined as the number of parasitized FAW larva evidenced by the presence of parasitoid eggs on their surface. The age-specific EPR (*k_x_*) was calculated following the standard procedures [56,57] as follows:

$$k_{x}=\frac{\sum_{j=1}^{\beta }S_{\mathrm{xj}}C_{\mathrm{xj}}}{\sum_{j=1}^{\beta }S_{\mathrm{xj}}}$$

where the age-stage-specific EPR (*C_xj_*) denotes the total number of parasitized FAW larva evidenced by the presence of parasitoid eggs contributed by individuals of age *x* and stage *j*. Incorporating *l_x_*, the age-specific net effective parasitism rate (*q_x_*) was calculated as:

*q_x_* = *l_x_k_x_*

The cumulative EPR (*C_x_*) denotes the total number of parasitized FAW larva evidenced by the presence of parasitoid eggs contributed by an average individual from birth to age *x*. The net EPR (*C*_0_) represents the total number of such parasitized larva produced by an average individual over its entire lifetime. These parameters were calculated using the following equations:

$$C_{x}=\sum_{i=0}^{x}l_{i}k_{i}\;\mathrm{and}\;C_{0}=\sum_{x=0}^{\infty }l_{x}k_{x}=\sum_{x=0}^{\infty }\sum_{j=1}^{\beta }S_{\mathrm{xj}}C_{\mathrm{xj}}$$

For a consistent comparison of EPR, it was integrated with finite rate to obtain the finite EPR (*ω*), following an established methodology [58,59] and calculated as follows:

$$\omega =\lambda \psi =\lambda \;\sum_{x=0}^{\infty }\sum_{j=1}^{\beta }a_{\mathrm{xj}}c_{\mathrm{xj}}$$

where *ψ* is the stable EPR and *a_xj_* represents the fractions of individuals at age *x* and stage *j* within the stable age-stage structure. The stable EPR was computed as:

$$\psi =\sum_{x=0}^{\infty }\sum_{j=1}^{\beta }a_{\mathrm{xj}}c_{\mathrm{xj}}$$

### 2.10. Non-Effective Parasitism Rate (NPR) Analysis

NPR quantifies the proportion of FAW larvae that were paralyzed by *Bracon hebetor* but showed no visible evidence of externally laid eggs on the host body. The age-specific NPR (*g*_x_) was calculated as follows:

$$g_{x}=\frac{\sum_{j=1}^{\beta }S_{\mathrm{xj}}d_{\mathrm{xj}}}{\sum_{j=1}^{\beta }S_{\mathrm{xj}}}$$

where *d_xj_* represents the age-stage-specific NPR for individuals at age *x* and stage *j*. Incorporating *l_x_*, the age-specific net NPR (*h_x_*) was calculated as:

*h_x_* = *l_x_g_x_*

The cumulative NPR (*N_x_*) denotes the total number of paralyzed FAW larva, without visible evidence of oviposition, contributed by an average individual from birth to age *x*. The net NPR (*N*_0_) represents the total number of such paralyzed larva produced by an average individual over its entire lifetime. These parameters were calculated using the following equations:

$$N_{x}=\sum_{i=0}^{x}l_{i}g_{i}\;\mathrm{and}\;N_{0}=\sum_{x=0}^{\infty }l_{x}g_{x}=\sum_{x=0}^{\infty }\sum_{j=1}^{\beta }S_{\mathrm{xj}}d_{\mathrm{xj}}$$

For a consistent comparison of NPR, it was integrated with a finite rate to obtain the finite NPR (*ε*) and calculated as follows:

$$\varepsilon =\lambda \gamma =\lambda \;\sum_{x=0}^{\infty }\sum_{j=1}^{\beta }a_{\mathrm{xj}}d_{\mathrm{xj}}$$

where *γ* is the stable NPR and was computed as:

$$\gamma =\sum_{x=0}^{\infty }\sum_{j=1}^{\beta }a_{\mathrm{xj}}d_{\mathrm{xj}}$$

### 2.11. Killing Rate (KR) Analysis

For an accurate assessment of a parasitoid’s biocontrol efficacy, both EPR and NPR must be considered, as each contributes to the overall host suppression potential. So, KR denotes the total number of FAW larva parasitized through EPR and NPR. The age-specific KR (*u_x_*) was computed as follows:

$$u_{x}=\frac{\sum_{j=1}^{\beta }S_{\mathrm{xj}}P_{\mathrm{xj}}}{\sum_{j=1}^{\beta }S_{\mathrm{xj}}}$$

where *P_xj_* denotes the age-stage-specific KR (i.e., *P_xj_* = *C_xj_* + *d_xj_*) of individuals at age *x* and stage *j*. Again, incorporating *l_x_*, the age-specific net KR (*w_x_*) was calculated as:

*w_x_* = *l_x_u_x_*

The cumulative KR (*Z_x_*) denotes the total number of parasitized FAW larva contributed by an average individual from birth to age *x*. The net KR (*Z*_0_) represents the total number of parasitized larvae produced by an average individual over its entire lifetime. These parameters were calculated using the following equations:

$$Z_{x}=\sum_{i=0}^{x}l_{i}u_{i}\;\mathrm{and}\;Z_{0}=\sum_{x=0}^{\infty }l_{x}u_{x}=\sum_{x=0}^{\infty }\sum_{j=1}^{\beta }S_{\mathrm{xj}}P_{\mathrm{xj}}=R_{0}+C_{0}+N_{0}$$

To enable a comprehensive comparison of KR, it was integrated with a finite rate to obtain the finite KR (*θ*) and calculated as follows:

$$\theta =\lambda \upsilon =\lambda \;\sum_{x=0}^{\infty }\sum_{j=1}^{\beta }a_{\mathrm{xj}}P_{\mathrm{xj}}$$

where *υ* is the stable KR and was computed as:

$$\upsilon =\sum_{x=0}^{\infty }\sum_{j=1}^{\beta }a_{\mathrm{xj}}P_{\mathrm{xj}}$$

The transformation rate (*Q_p_*), defined as the mean number of FAW larva that must be parasitized to produce a single parasitoid offspring, was computed as:

$$Q_{p}=\frac{Z_{0}}{R_{0}}=\frac{R_{0}+C_{0}+N_{0}}{R_{0}}$$

### 2.12. Population Growth and Killing Rate Projection

Population and killing rate projections were started with 10 *B. hebetor* eggs reared on two *S. frugiperda*–host complexes. Simulations were extended over 60 days under the assumption of unrestricted resource availability and absence of biotic and abiotic constraints, thereby enabling an unbiased assessment of killing potential. Simulations were projected by using the TIMING-MS Chart [60], following the established methodology [51,61]. The projected population size and killing potential at time *t* were estimated as follows:

$$N\left(t\right)=\sum_{j=1}^{\beta }\sum_{x=0}^{\infty }n_{\mathrm{xj},t}\;\mathrm{and}\;P\left(t\right)=\sum_{j=1}^{\beta }\sum_{x=0}^{\infty }{n_{\mathrm{xj}}c}_{\mathrm{xj},t}$$

where *n_xj_*_,*t*_ represents the number of individuals at age *x* and stage *j* at time *t* [62].

### 2.13. Functional Response Analysis

The analysis of functional response involved two main steps: determining the response type and estimating its parameters [63]. Logistic regression was used to examine the proportion of parasitized hosts relative to initial density, fitting a polynomial model:

$$\frac{N_{a}}{N_{0}}=\frac{\exp(P_{0}+P_{1}N_{0}+P_{2}N_{0}^{2}+P_{3}N_{0}^{3})}{1+\exp(P_{0}+P_{1}N_{0}+P_{2}N_{0}^{2}+P_{3}N_{0}^{3})}$$

where, *N_a_* is the number of parasitized FAW larva, *N*_0_ is initial host density, and *P*_0_, *P*_1_, and *P*_2_ are the intercept, linear, and quadratic coefficients, respectively. A negative *P*_1_ indicates a type II response, whereas a positive *P*_1_ with negative *P*_2_ indicates type III. For type II responses, handling time (*T_h_*) and search efficiency (*a*) were estimated using Holling’s disc equation [64] and Rogers’ random attack model [65] as follows:

$$\text{Hollings’s disc equation}:N_{a}=\frac{\mathrm{aT}N_{0}}{1+aT_{h}N_{0}}$$

Roger’s random attack equation: *N_a_* = *N*_0_ {1 − exp[*a*(*T_h_N*_0_ − *T*)]}

where *T* is the total exposure time (24 h), *N*_0_ is the initial host number, and *N_a_* is the number of parasitized larvae.

### 2.14. Statistical Analysis

Statistical comparisons were performed at 5% significance level based on confidence intervals to assess differences between treatments. The standard errors (SE) and variances of all the estimated parameters were obtained using a bootstrap resampling procedure with 100,000 iterations [66]. All graphical outputs were prepared using SigmaPlot version 15. First, the homogeneity and normality of variances of the parasitism data collected across host densities for each host plant were examined. Once the presumptions were satisfied, one-way ANOVA followed by Tukey’s HSD test was used to compare mean parasitism among densities. Functional response curves were generated using the R software (version 4.6.1). Greenhouse experiment data on EPR, NPR, and KR were analyzed using independent *t*-tests in the R software (package: stats) [67], withsignificance determined at *p* < 0.05.

## 3. Results

### 3.1. Host-Plant-Mediated Effects on Developmental Periods and Reproductive Parameters of B. hebetor

The developmental durations of *B. hebetor* varied significantly (*p* < 0.05) across the two host plants on which FAW larvae were reared. The mean duration of each developmental stage, adult longevity and total longevity are summarized in Table 1. *Bracon hebetor* developing on maize-fed FAW recorded significantly shorter egg, larval, pupal and pre-adult periods (2.28 ± 0.05, 3.35 ± 0.06, 6.44 ± 0.09 and 12.18 ± 0.13 days, respectively) than those developed on castor-fed larvae (2.85 ± 0.04, 3.94 ± 0.03, 7.06 ± 0.11, and 14.06 ± 0.11 days, respectively). Adult longevity also differed significantly among host plants, with adults emerging from maize-fed hosts living significantly longer (31.11 ± 0.36 days) than those from castor-fed hosts (29.35 ± 0.37 days) in both sexes. The total lifespan of *B. hebetor* showed similar host-plant-mediated effects, with slightly greater longevity in both the sexes emerging from maize-fed FAW larvae (Table 1). Of the initial 100 eggs used per treatment, 68% (castor) and 75% (maize) successfully hatched. Finally, 48 (28 females and 20 males) and 55 (33 females and 22 males) adults emerged from FAW–castor and FAW–maize, respectively (Table 1).

Reproductive parameters of *B. hebetor* varied significantly (*p* < 0.05) between the two host plant systems (Table 2). The adult pre-oviposition period (APOP) did not differ significantly between FAW–castor and FAW–maize. However, *B. hebetor* females from maize-fed FAW exhibited a shorter total pre-oviposition period (TPOP) (13.03 ± 0.23 days) as compared to castor-fed FAW (14.60 ± 0.11 days). The oviposition period was also longer on maize-fed hosts (32.58 ± 0.22 days) in contrast to castor-fed hosts (30.75 ± 0.14 days). Similarly, fecundity was significantly higher in females reared on FAW–maize (247.09 ± 0.41 eggs) in comparison to FAW–castor (226.50 ± 0.86 eggs).

### 3.2. Host-Plant-Mediated Effects on Population Parameters of B. hebetor

The population parameters of *B. hebetor* parasitizing on the two *S. frugiperda*–host plant systems are summarized in Table 3. *Bracon hebetor* developed on maize-fed FAW showed significantly higher intrinsic rate of increase (*r* = 0.217 ± 0.008 d^−1^) and finite rate of increase (*λ* = 1.242 ± 0.011 d^−1^) than those on castor-fed FAW (*r* = 0.186 ± 0.008 d^−1^ and *λ* = 1.205 ± 0.010 d^−1^, respectively). The FAW–maize host plant complex also showed a numerically higher net reproductive rate (*R*_0_ = 81.54 ± 11.551 offspring) and gross reproductive rate (*GRR* = 160.85 ± 15.129 offspring) than FAW–castor (*R*_0_ = 63.42 ± 10.182 and *GRR* = 145.05 ± 15.172 offspring), but these differences were not statistically significant. Mean generation time (*T*) was significantly shorter on maize-fed FAW (20.263 ± 0.289 days) as compared to castor-fed FAW (22.241 ± 0.205 days), whereas doubling time was slightly longer on castor-fed hosts (3.714 ± 0.178 days) than on maize-fed hosts.

**Figure 1:**
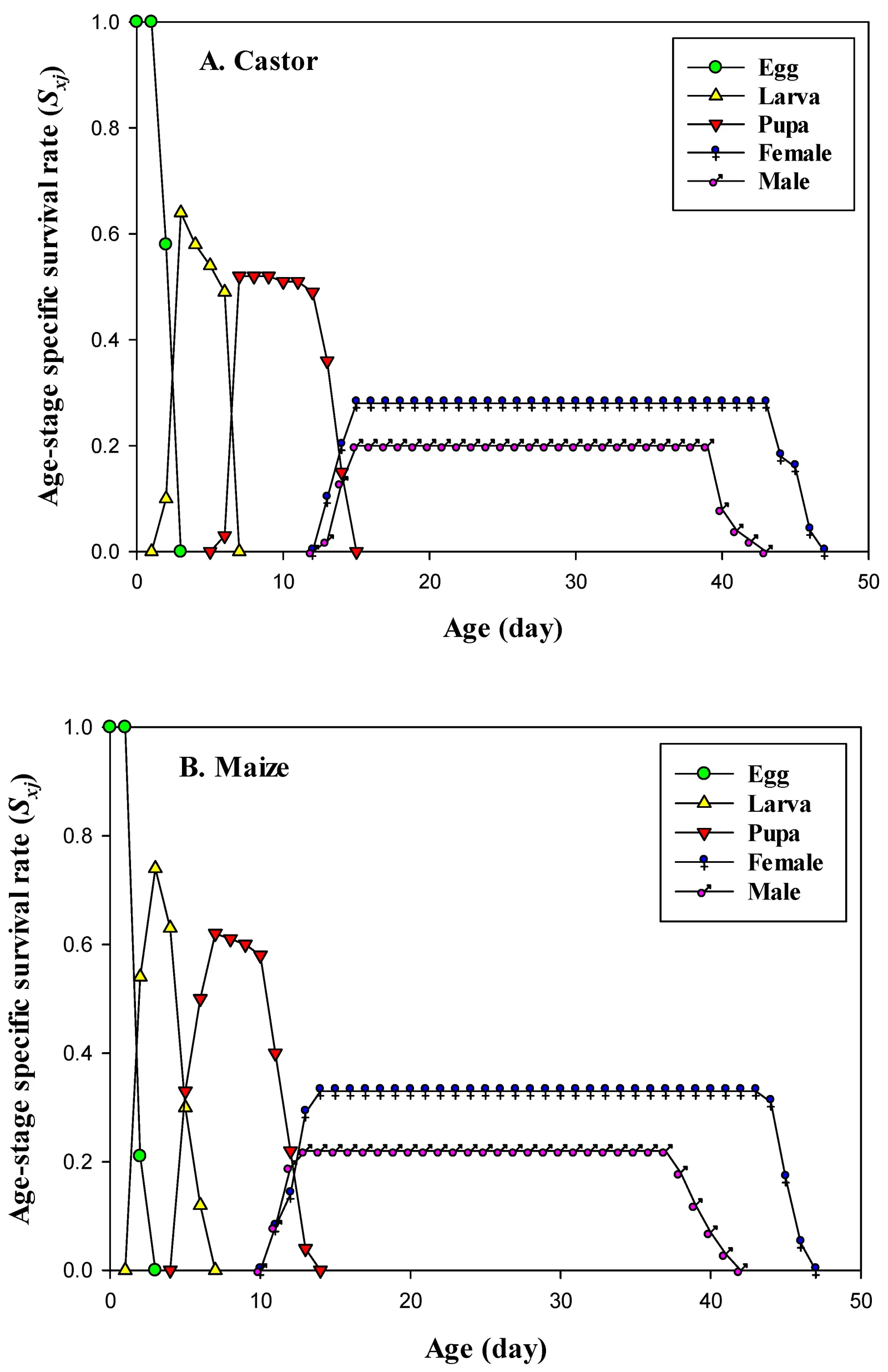
Age-stage-specific survival rates (*S_xj_*) of *Bracon hebetor* parasitizing on *Spodoptera frugiperda* on castor (**A**) and maize (**B**).

The age-stage-specific survival rate (*S_xj_*) of *B. hebetor* developing from FAW larva on the two host plants is depicted in Figure 1. The probability of reaching adulthood was higher for parasitoids reared on FAW–maize, with survival rates of 0.22 for males and 0.33 for females. In comparison, survival to adulthood on FAW–castor was 0.20 and 0.28 for males and females, respectively. The overlapping survival curves across developmental stages reflect individual variation in developmental progression within each host plant system.

**Figure 2:**
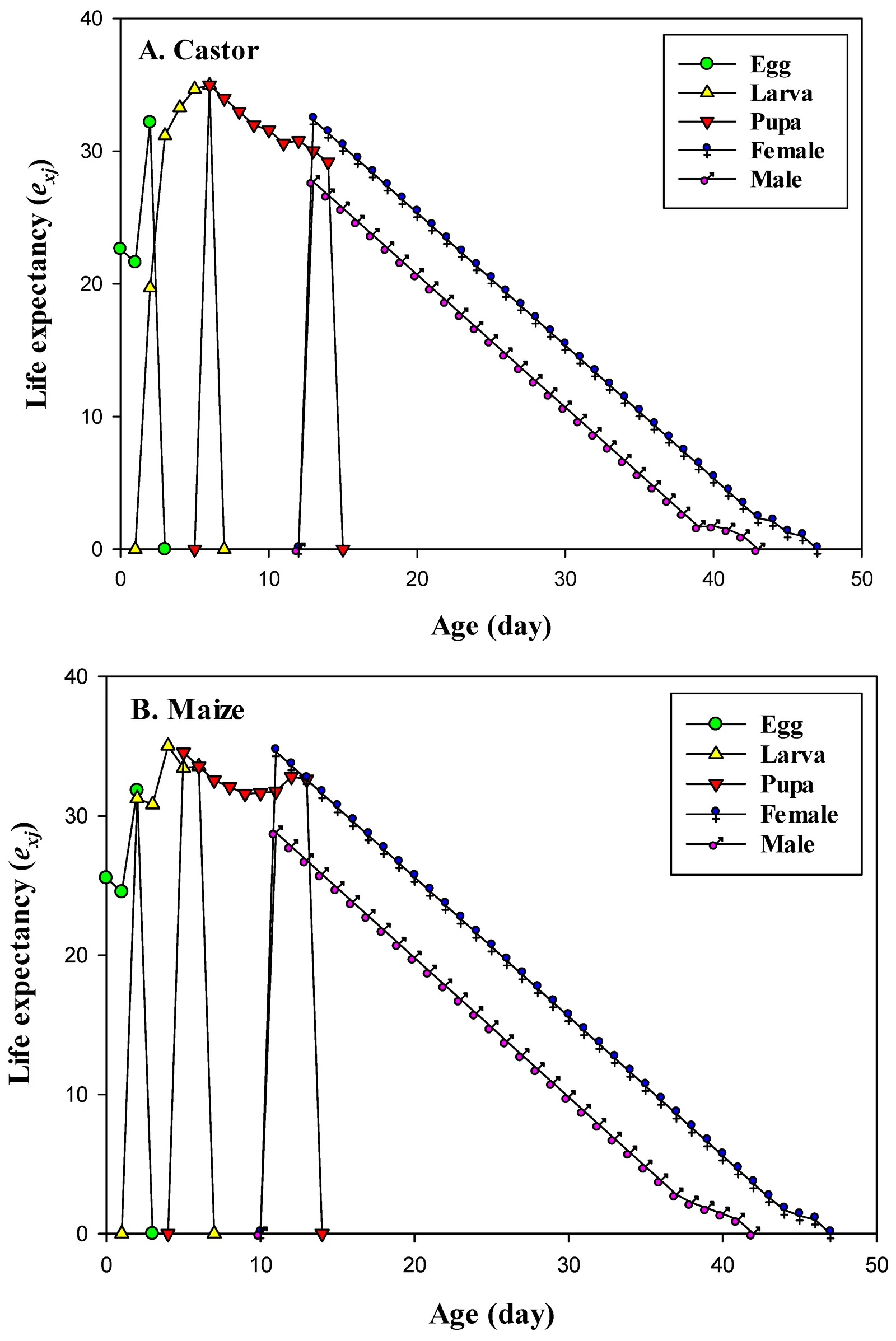
Age-stage-specific life expectancies (*e_xj_*) of *Bracon hebetor* parasitizing on *Spodoptera frugiperda* on castor (**A**) and maize (**B**).

The age-stage-specific life expectancy (*e_xj_*) of *B. hebetor* developed on FAW larva reared on castor and maize is shown in Figure 2. The *e_xj_* of a newly laid egg was higher on maize-fed FAW (25.54 days) as compared to castor-fed FAW (22.63 days). Adult females also exhibited greater longevity when developed on maize-fed FAW, with a peak *e_xj_* of 34.60 days at age 11 day in contrast to castor-fed FAW (32.35 days at age 13 day). Life expectancy declined steadily with advancing stage due to the absence of field-related stressors in the laboratory setting.

**Figure 3:**
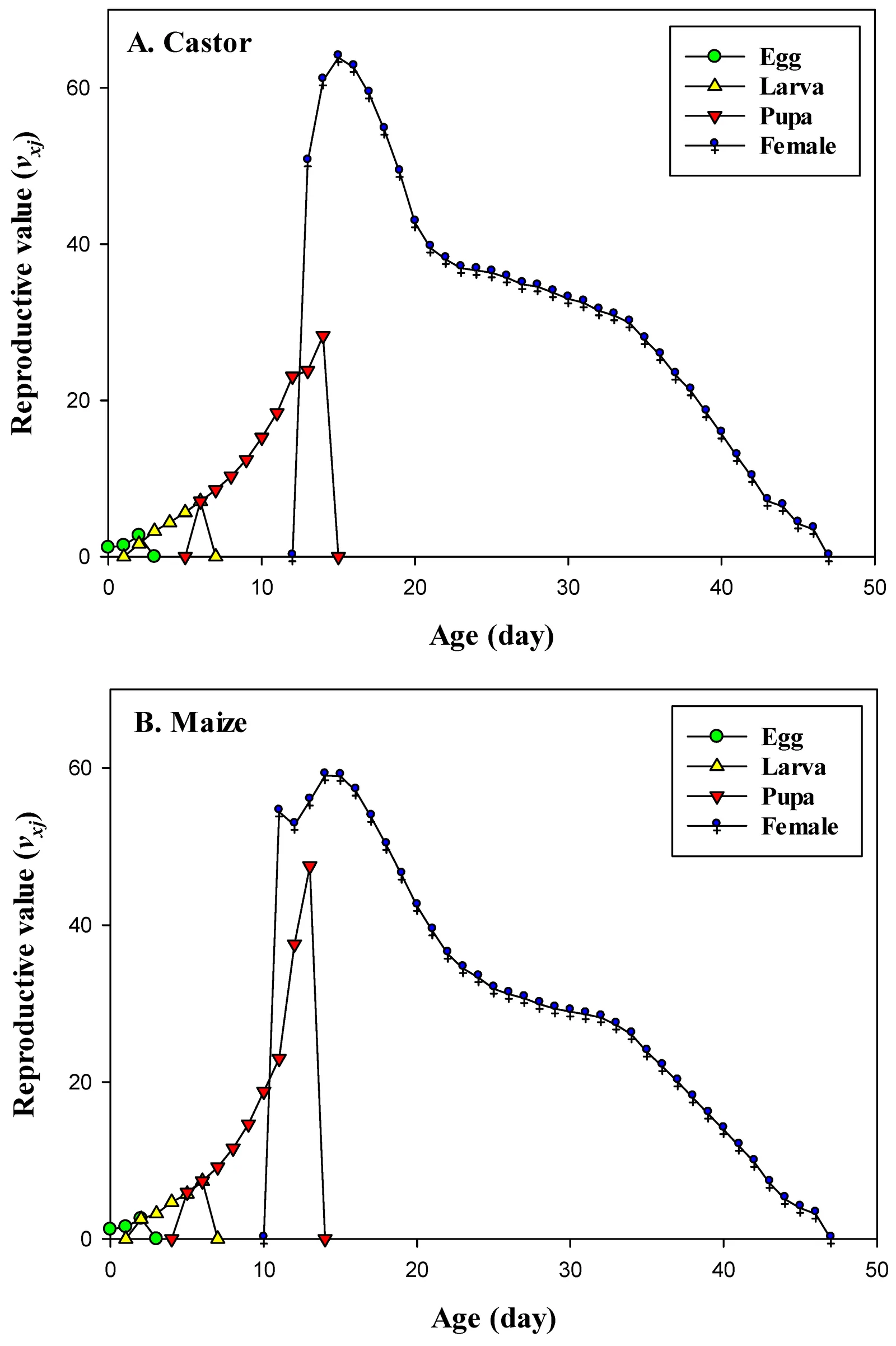
Age-stage-specific reproductive values (*v_xj_*) of *Bracon hebetor* parasitizing on *Spodoptera frugiperda* on castor (**A**) and maize (**B**).

The age-stage-specific reproductive values (*v_xj_*) of *B. hebetor* developed on FAW larva reared on castor and maize are illustrated in Figure 3. The *v_xj_* at emergence, which corresponds to the finite rate of increase, was higher on maize-fed hosts (1.242 d^−1^) in contrast to castor-fed hosts (1.205 d^−1^). The peak *v_xj_* value was slightly lower and occurred earlier on maize-fed hosts, reaching 59.02 d^−1^ on day 14, compared with 63.88 d^−1^ on day 15 for castor-fed hosts. The shift in the peak to a later age on FAW–castor reflected a slower progression to peak reproductive capacity.

**Figure 4:**
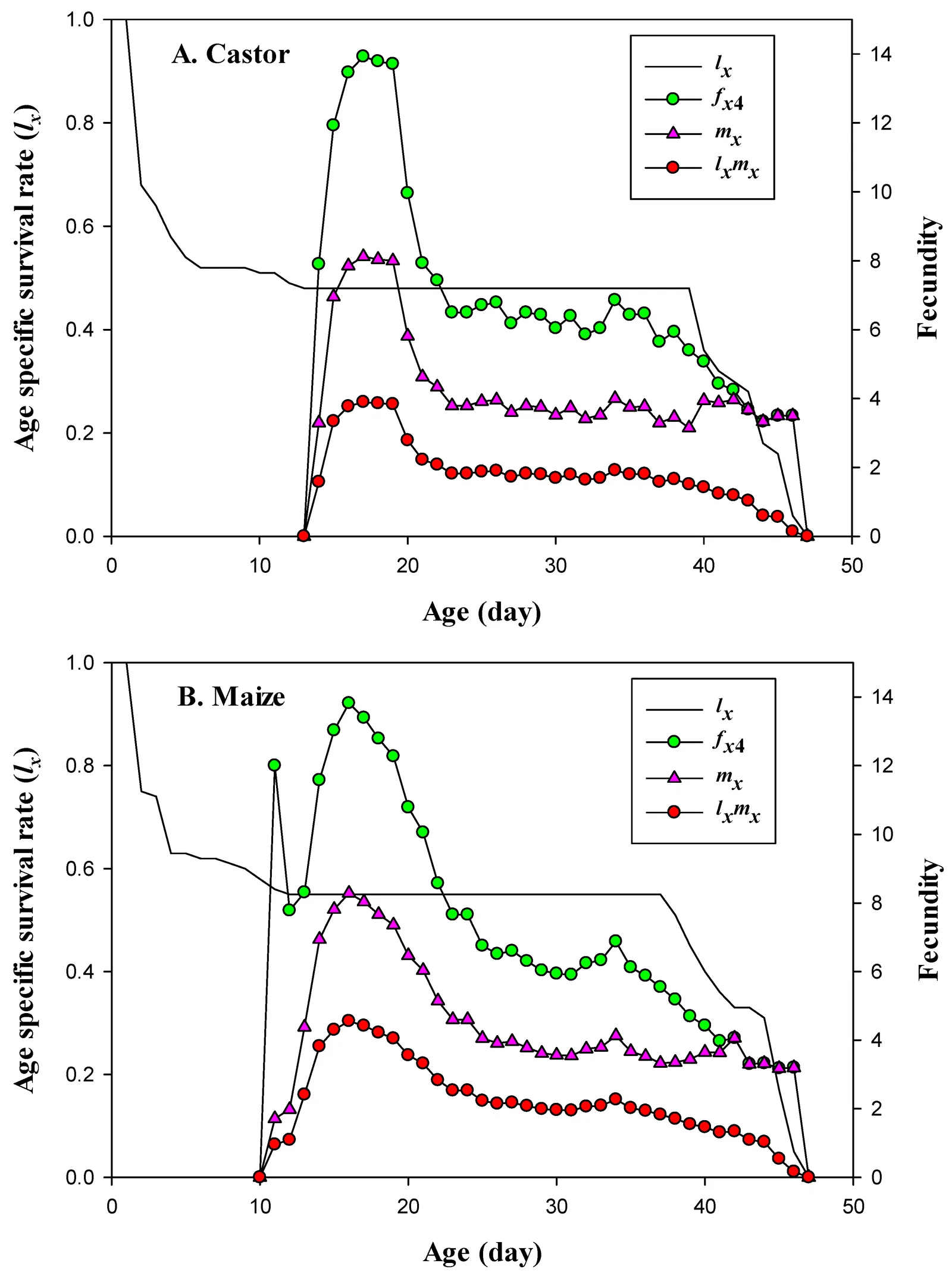
Age-specific survival rates (*l_x_*), fecundities (*m_x_*), maternities(*l_x_m_x_*) and age-stage specific fecundities (*f_x_*_4_) of *Bracon hebetor* parasitizing on *Spodoptera frugiperda* on castor (**A**) and maize (**B**).

The age-specific survival rates (*l_x_*), age-specific fecundities (*m_x_*), age-stage-specific fecundities (*f_x_*_4_) and age-specific maternities (*l_x_m_x_*) of *B. hebetor* parasitizing on FAW larva reared on maize and castor are displayed in Figure 4. *Bracon hebetor* reared on FAW–maize reached their highest *l_x_m_x_* peak of 4.56 on day 16, whereas those reared on castor-fed larvae attained their maximum *l_x_m_x_* value of 3.90 on day 17.

### 3.3. Host-Plant-Mediated Effects on Effective Parasitism Rate, Non-Effective Parasitism Rate, and Killing Rate of B. hebetor Under Laboratory and Semi-Field Conditions

**Figure 5:**
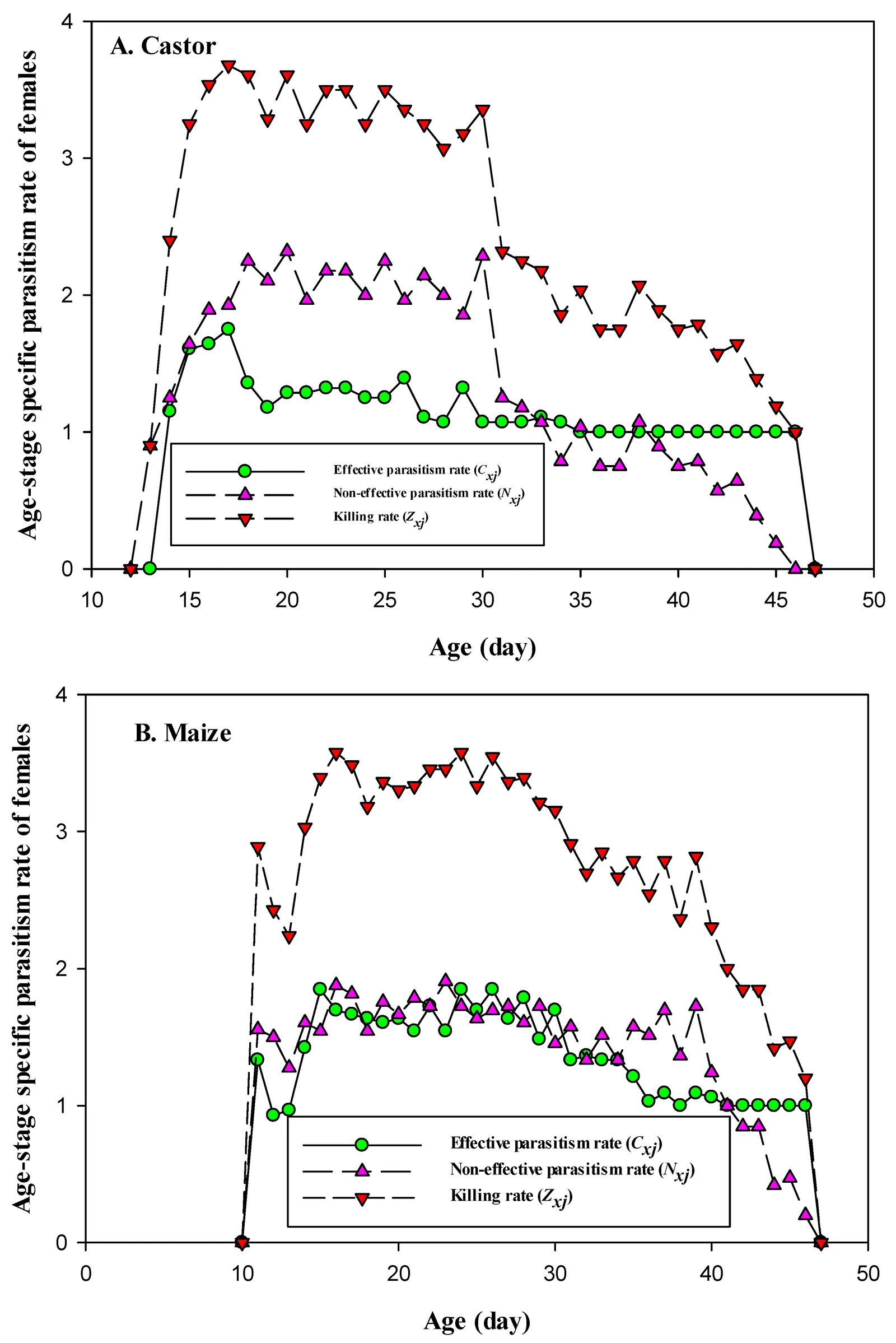
Age-stage-specific parasitism rates of *Bracon hebetor* parasitizing on *Spodoptera frugiperda* on castor (**A**) and maize (**B**), with age counted from birth.

**Figure 6:**
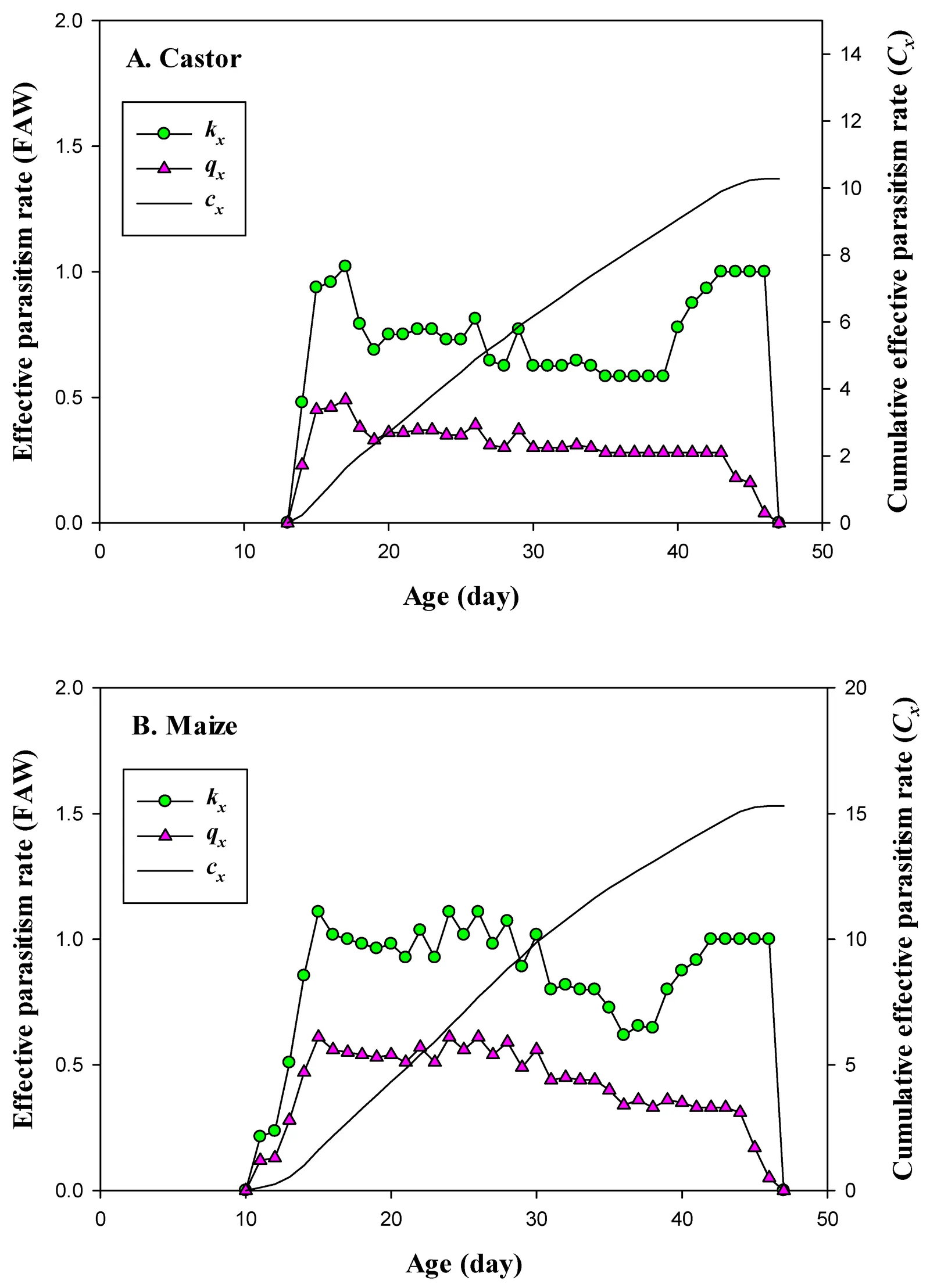
Age-specific effective parasitism rates (*k_x_*), age-specific net effective parasitism rates (*q_x_*) and cumulative effective parasitism rates (*C_x_*) of *Bracon hebetor* parasitizing on *Spodoptera frugiperda* on castor (**A**) and maize (**B**), with age counted from birth.

**Figure 7:**
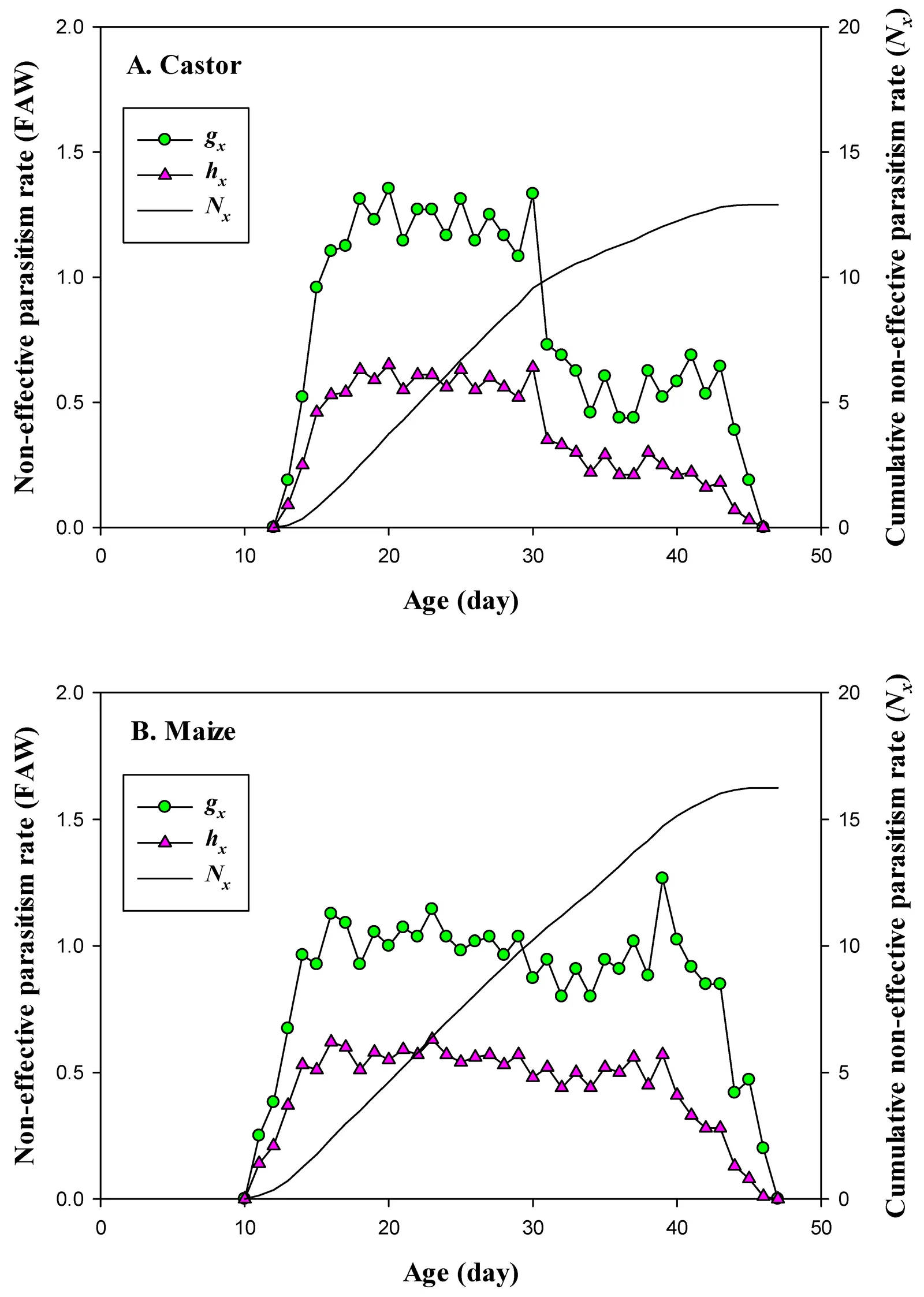
Age-specific non-effective parasitism rates (*g_x_*), age-specific net non-effective parasitism rates (*h_x_*) and cumulative non-effective parasitism rates (*N_x_*) of *Bracon hebetor* parasitizing on *Spodoptera frugiperda* on castor (**A**) and maize (**B**), with age counted from birth.

**Figure 8:**
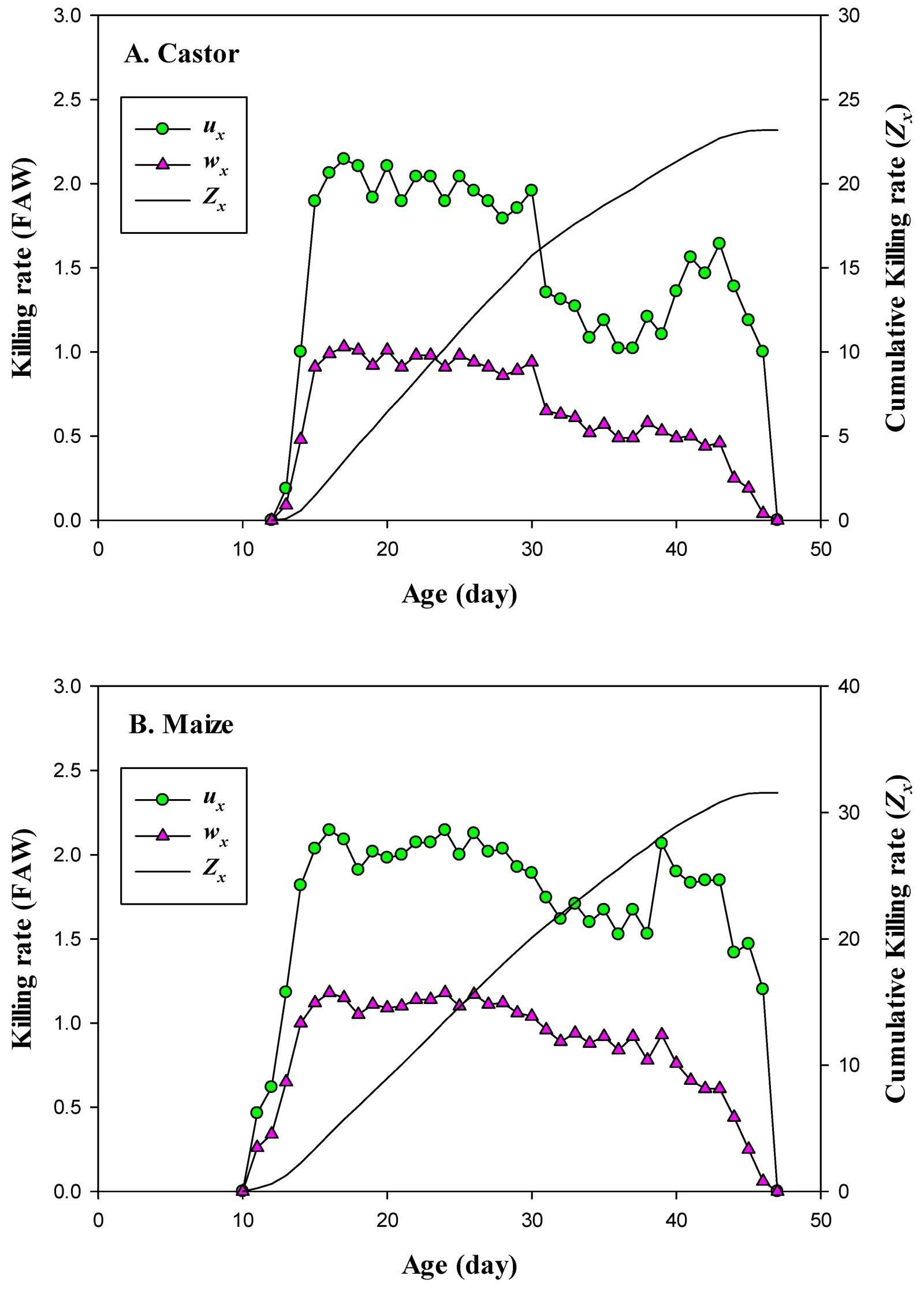
Age-specific killing rates (*u_x_*), age-specific net killing rates (*w_x_*) and cumulative killing rates (*Z_x_*) of *Bracon hebetor* parasitizing on *Spodoptera frugiperda* on castor (**A**) and maize (**B**), with age counted from birth.

The *C_xj_*, *N_xj_*, and *Z_xj_* values of *B. hebetor* females developed on the two FAW–host plant systems are presented in Figure 5, showing the average number of larvae parasitized by a female at a specific age *x*. All three curves initially increased, reaching medium peaks during the mid-reproductive period, and subsequently declined at later stages. Figure 6, Figure 7 and Figure 8 depict the EPR, NPR, and KR of *B. hebetor* with their corresponding cumulative rates, respectively. The *k_x_*, *g_x_*, and *u_x_* of *B. hebetor* developed on maize-fed FAW (1.10, 1.35, and 2.14 parasitized larva at 15, 20, and 16 days, respectively) were higher than castor-fed FAW (1.02, 1.26, and 2.10 parasitized larva at 17, 39, and 17 days, respectively). A similar trend was observed for the *q_x_*, *h_x_*, and *w_x_* of *B. hebetor* when developed on maize-fed FAW in comparison to castor-fed FAW. Host plant significantly (*p* < 0.05) influenced the total effective parasitism rate (EPR) and killing rate (KR) of females, with higher rates on maize-fed hosts (EPR = 46.393 ± 0.989; KR = 95.636 ± 0.832) than on castor-fed hosts (EPR = 36.714 ± 0.793; KR = 82.785 ± 1.132). In contrast, the total non-effective parasitism rate (NPR) did not differ significantly between maize-fed (49.242 ± 1.999) and castor-fed hosts (46.071 ± 1.906) (Table 4). Further, the net, stable, and finite parasitism and killing indices revealed clear host-plant-mediated differences (Table 4). A similar trend was recorded for the net, stable, and finite parasitism and killing indices, which were higher on maize-fed FAW than on castor-fed FAW (Table 4). Further, *Q_p_* was higher on maize-fed FAW (1.387 ± 0.06) as compared to castor-fed FAW (1.365 ± 0.09).

**Figure 9:**
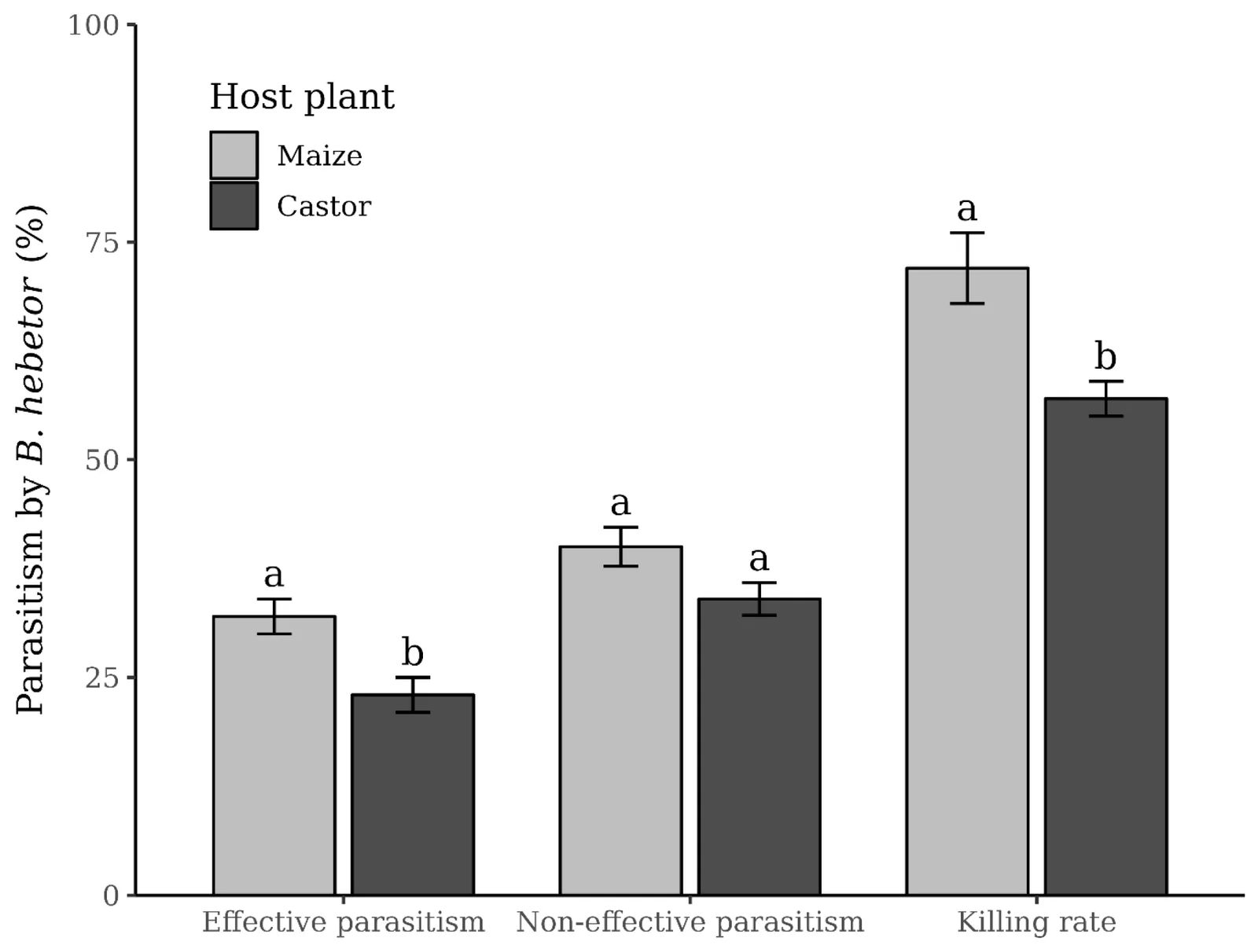
Percentage parasitism by *Bracon hebetor* parasitizing on *Spodoptera frugiperda* larvae on maize and castor under greenhouse conditions. Effective parasitism, non-effective parasitism, and killing rate are shown as mean ± SE. Different letters above bars indicate statistically significant differences between host plants for each parameter (*p* < 0.05, independent *t*-test).

Further, the greenhouse experiment indicated host-plant-mediated differences in the parasitism efficiency of *B. hebetor*. The effective parasitism rate was significantly higher on maize-fed larvae (32%) compared with castor-fed larvae (23%) (Figure 9; df = 1,8; *p* < 0.05; t = 3.182). Non-effective parasitism did not vary significantly (df = 1,8; *p* = 0.079, t = 2.058) between the host plants but was relatively higher when recorded on maize (40%) than castor (34%). In contrast, the killing rate was higher on maize-fed larvae (~72%) and lower on castor-fed larvae (~57%) (Figure 9; df = 1,8; *p* < 0.05, t = 3.313).

### 3.4. Host-Plant-Mediated Effects on Population Growth and Killing Rate Projection of B. hebetor

**Figure 10:**
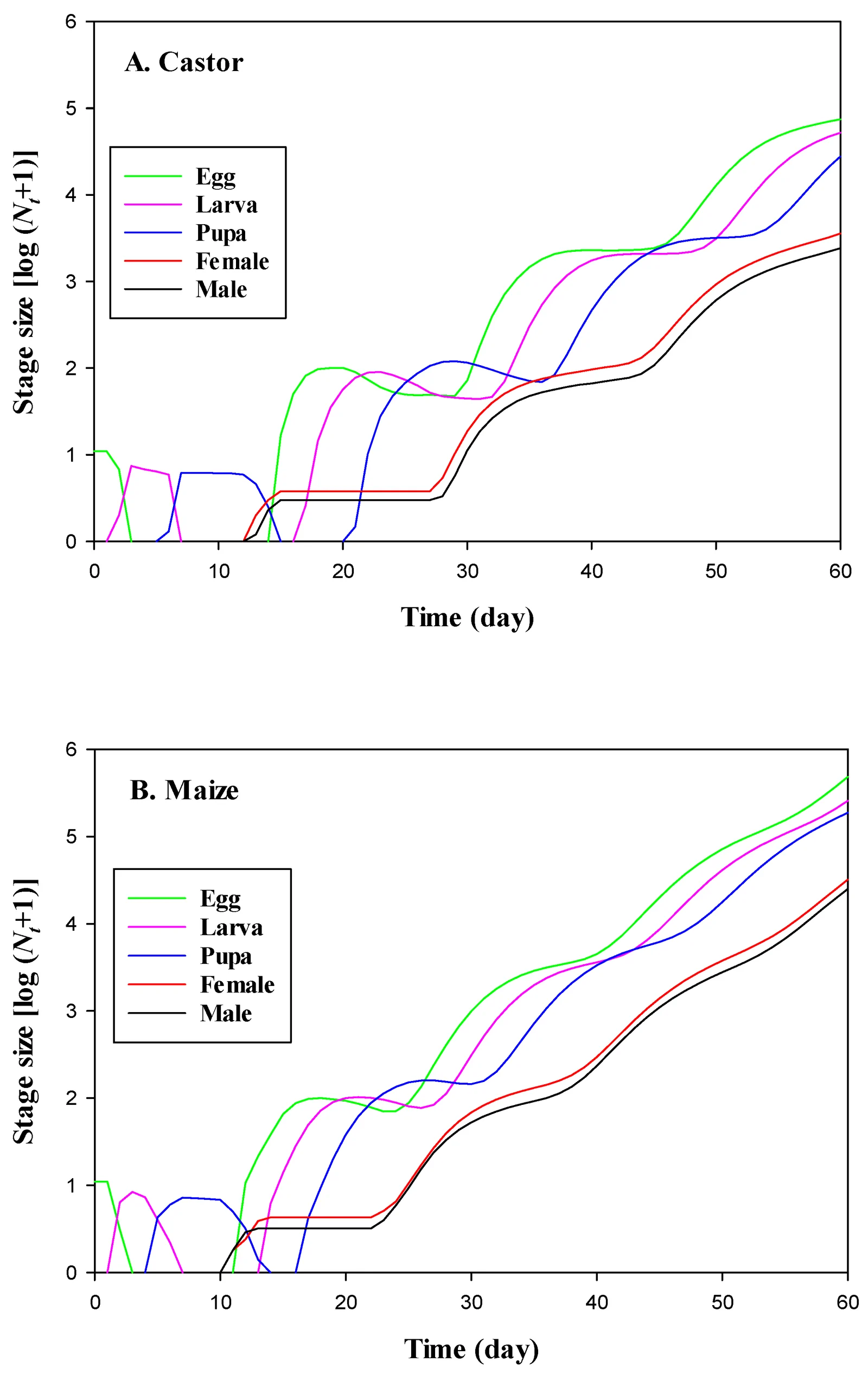
Stage structure and population growth of *Bracon hebetor* parasitizing on *Spodoptera frugiperda* on castor (**A**) and maize (**B**).

**Figure 11:**
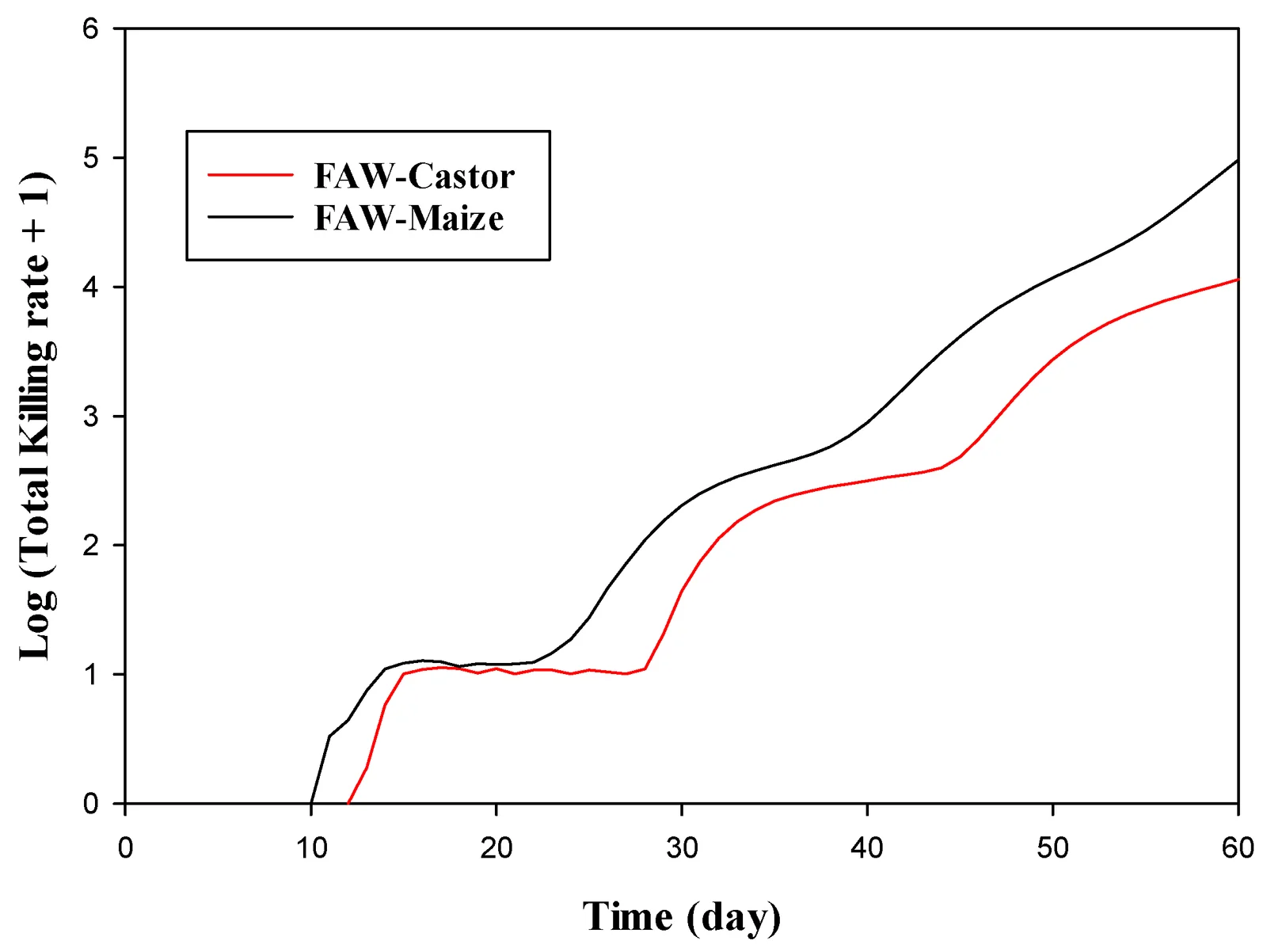
Projection of total killing rates of *Bracon hebetor* parasitizing on *Spodoptera frugiperda* on castor and maize.

Using the life history, survival and fecundity estimated from the age-stage, two-sex life table, the projected population growth and stage-specific structure of *B. hebetor* developed on FAW larvae reared on maize and castor are illustrated in Figure 10. The fluctuations among stages are reflected in the simulations, as the age-stage, two-sex model distinguishes overlapping developmental stages. Beginning with an initial cohort of ten newly laid eggs, *B. hebetor* showed the fastest population expansion when reared on maize-fed FAW by day 60. Population buildup of maize-fed hosts was consistently higher as compared to castor-fed hosts (Figure 10). Correspondingly, the total killing rate of *B. hebetor* was greater on maize-fed FAW larvae in contrast to castor-fed FAW throughout the simulation period (Figure 11).

### 3.5. Host-Plant-Mediated Effects on Functional Response of B. hebetor

**Figure 12:**
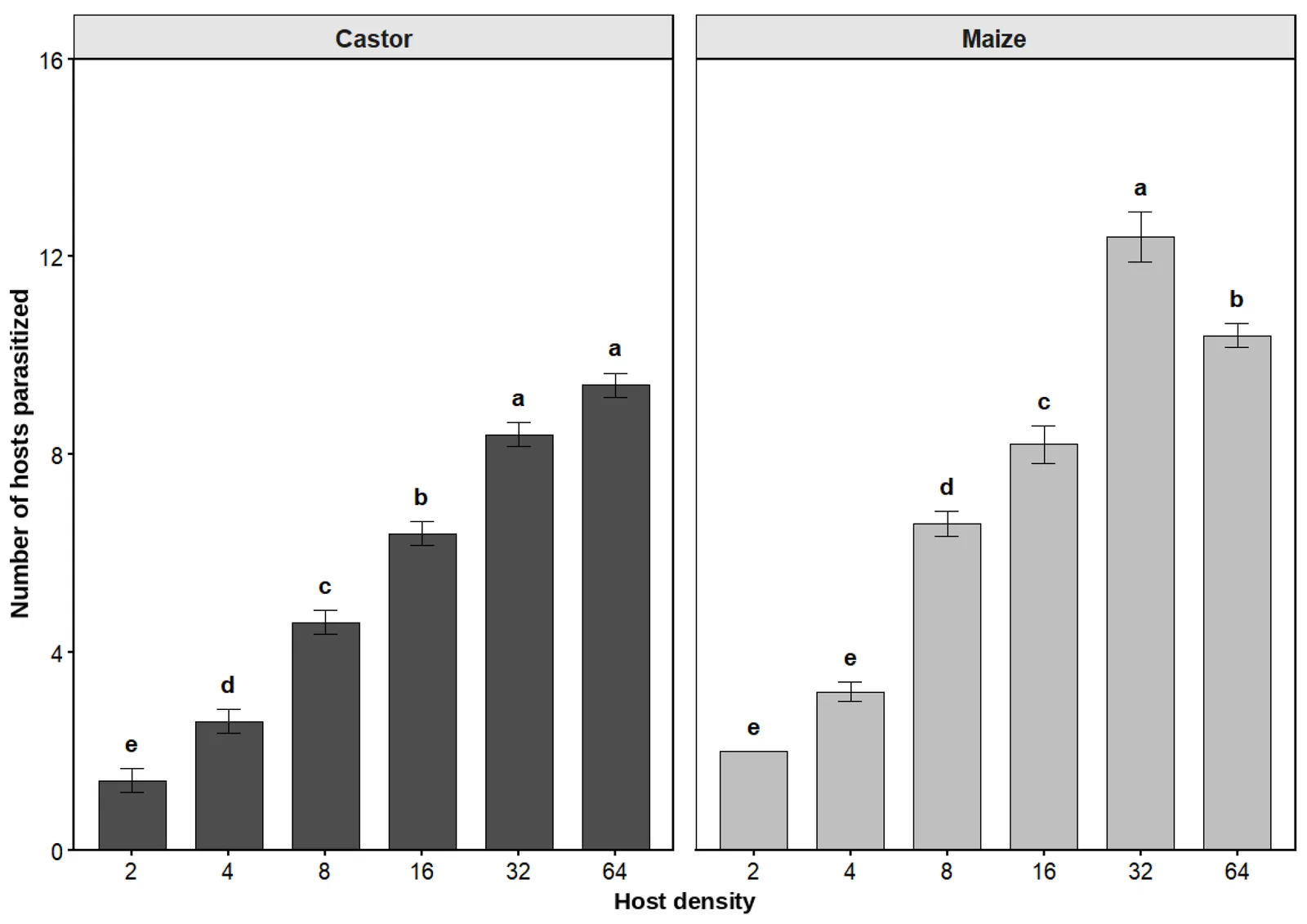
Number of hosts parasitized by *Bracon hebetor* against the different densities of *Spodoptera frugiperda* on castor and maize. Values in the bar diagram are mean ± SE, and different letters above the standard error bars indicate significant differences by Tukey’s HSD (*p* < 0.05) between different densities of *Spodoptera frugiperda*.

The functional response of *B. hebetor* was evaluated across a range of densities of fifth-instar FAW larvae on two host plants, maize and castor. Significant differences in parasitism rates were observed across host densities for larvae reared on both maize (df = 5,24; F = 174.971; *p* < 0.01) and castor (df = 5,24; F = 168.178, *p* < 0.01) (Figure 12). Logistic regression analysis further showed that the parasitoid exhibited a typical type II functional response on both host plants, as evidenced by the negative linear coefficients (Table 5).

**Figure 13:**
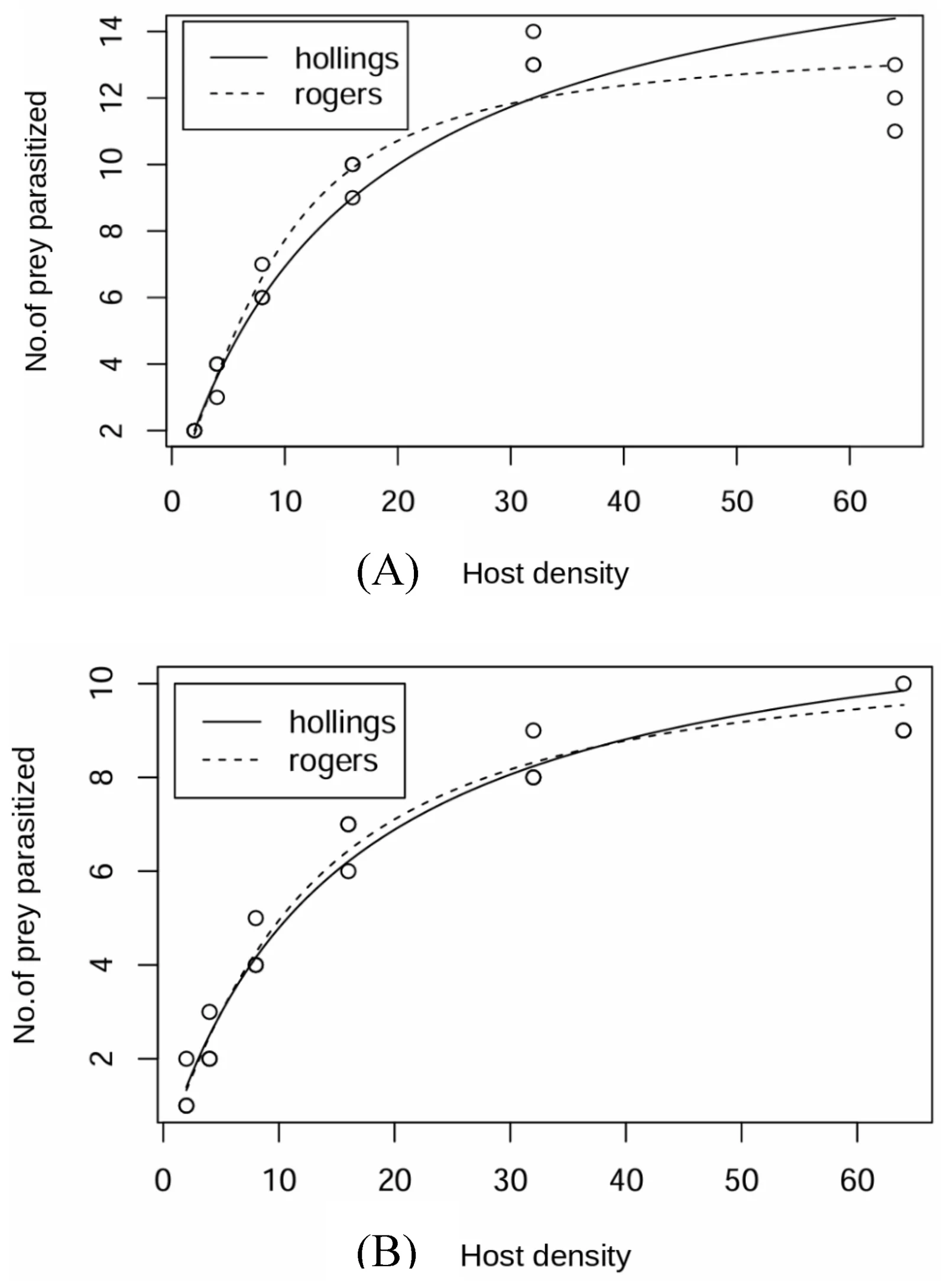
Functional response of *Bracon hebetor* to different densities of *Spodoptera frugiperda* when subjected to Holling’s and Roger’s models. Fitted lines exhibited decelerating curves, indicating a type II functional response. (**A**) Fitting of functional response curve of *B. hebetor* parasitizing *Spodoptera frugiperda* on maize; (**B**) fitting of functional response curve of *B. hebetor* parasitizing *Spodoptera frugiperda* on castor.

Application of Holling’s and Roger’s models indicated that the parasitism rate by *B. hebetor* increased with larval density until reaching an asymptote, consistent with saturation effects (Figure 13). Both models were used to estimate the functional response parameters for *B. hebetor* on each host plant. Handling time (*T_h_*) and searching efficiency (*a*) estimates are presented in Table 6. According to Holling’s disc equation, the searching efficiency on castor-fed FAW (0.7869 ± 0.142 h^−1^) was lower in comparison to maize-fed FAW (1.1252 ± 0.001 h^−1^). Conversely, Roger’s model showed higher searching efficiency on maize-fed hosts (3.3243 ± 0.872 h^−1^) as compared to castor-fed hosts (1.2464 ± 0.346 h^−1^). Handling times estimated by both models were slightly longer in Roger’s model than in Holling’s, with values on castor-fed hosts being 0.0817 ± 0.014 days (Holling) and 0.0912 ± 0.015 days (Roger) and on maize-fed hosts 0.0556 ± 0.000 days (Holling) and 0.0718 ± 0.008 days (Roger). Maximum parasitization rates (T/T_h_) from Holling’s model were calculated as 12.239 larvae on castor-fed FAW and 17.985 larvae on maize-fed FAW. Roger’s model yielded maximum parasitization rates of 10.964 (castor-fed FAW) and 13.927 (maize-fed FAW). Model fit comparisons using the Akaike information criterion (AIC) indicated that Holling’s model best described the functional response on castor-fed larvae (AIC = 56.57 vs. 57.27 for Roger’s), while Roger’s model provided a better fit on maize-fed larvae (AIC = 51.32 vs. 52.30 for Holling’s) (Table 6).

## 4. Discussion

The present study indicates that host plant identity influenced the developmental biology and demographic performance of *B. hebetor* reared on FAW under the experimental conditions. These findings are consistent with the extensive literature indicating that plant nutritional quality, secondary metabolites, and allelochemical profiles impose bottom-up constraints on herbivores, subsequently modulating parasitoid growth, physiology, and fitness [28,68,69,70]. Similar findings suggest that the performance of natural enemies may be influenced by the morphological, physical, chemical and nutritional characteristics of the host plant on which the herbivore develops [71,72].

Age, size and host species are recognized determinants of parasitoid suitability, influencing developmental periods and overall fitness [73,74]. Host plants and their varieties differ in nutrient composition, not only in the quality and quantity of nutrients but also in the accessibility of key minerals and other dietary components [75,76]. Maize is generally considered a highly suitable host for FAW, supporting rapid larval development and greater pupal biomass [4]. In contrast, castor is chemically defended, containing ricin, ricinine and diverse phenolic compounds that can impair herbivore growth and nutrient assimilation [43,44]. Larvae feeding on castor may therefore differ in nutritional composition and accumulate defensive metabolites, potentially diminishing their suitability for parasitoid development. Such differences among host plants may arise from a combination of extrinsic factors, including nutrient content and secondary metabolites, and intrinsic genetic traits of the host plants [77,78]. However, as these biochemical attributes were not quantified in the present study, these interpretations remain inferential and should be validated in further investigations. Evidence on plant-mediated influences on *B. hebetor* in the FAW system remains scarce; however, comparable patterns have been reported in other host–parasitoid interactions. For example, *Aphelinus asychis* (Walker, 1839) (Hymenoptera: Aphelinidae) developed faster and showed longer longevity, higher fecundity and a longer oviposition period on *Myzus persicae* (Sulzer, 1776) (Hemiptera: Aphididae) reared on chili pepper than on cabbage [79]. Likewise, life-history traits of *Habrobracon hebetor* (Say, 1836) (Hymenoptera: Braconidae) were influenced when parasitizing *Spodoptera exigua* (Hübner, 1808) (Lepidoptera: Noctuidae) larvae raised on different sugar beet genotypes [80].

Reproductive performance also differed between host plants: females from maize-fed larvae had higher fecundity and a shorter TPOP, whereas APOP did not differ significantly. Higher reproductive output on more suitable hosts has been reported in parasitoids and is consistent with the idea that suitable hosts allow for greater allocation of resources to reproduction [79]. This is also consistent with the broader view that plant functional traits and secondary metabolites can shape multitrophic interactions by influencing herbivore suitability and, in turn, the fitness of associated natural enemies [29,81]. Demographic analyses showed higher *r* and λ and a shorter *T* on maize-fed FAW, whereas *R*_0_ and GRR did not differ significantly. The reproductive value peaked earlier on maize-fed hosts, which may reflect faster reproductive maturation; early reproduction may be advantageous under fluctuating field conditions [82]. A shorter generation time may also allow for faster population turnover of the parasitoid, which could help it keep pace with FAW population growth, although this was not tested here. Similar host-plant-mediated demographic effects have been reported in other systems, including *Diadegma semiclausum* Hellen, 1949 (Hymenoptera: Ichneumonidae) on crucifer-feeding hosts [31], *A. asychis* on aphid–plant complexes [79] and *H. hebetor* on beet armyworm [80].

Integrating life table analysis with parasitism metrics is essential for accurately estimating parasitoid potential in bio-control programmes [83]. These results provide quantitative evidence that host-plant-mediated effects can influence the parasitism potential of *B. hebetor* when reared on FAW. Parasitoids reared on maize-fed larvae exhibited higher effective parasitism and killing rates (*Z*_0_), along with greater conversion efficiency (*Qp*) and consistently elevated parasitism parameters, indicating enhanced suppression potential. Such differences may reflect variation in host suitability, although host nutritional quality was not measured, enabling faster and more effective pest regulation, particularly during early crop stages. Additionally, increased non-effective parasitism on maize-fed hosts is noteworthy, as venom-induced paralysis alone can result in host mortality in this idiobiont ectoparasitoid, thereby contributing to overall control impact [84]. These findings align with previous studies on *H. hebetor* on *Ephestia elutella* Hubner, 1796 (Lepidoptera: Pyralidae) [85,86] and *C. cephalonica* [41], supporting the broader ecological concept that host plant quality influences parasitoid fitness by modulating herbivore suitability [79,87].

Our results showed that *B. hebetor* exhibited a type II functional response to FAW larva on both castor and maize, consistent with parasitoids whose foraging is constrained by handling time at higher host densities [88]. However, the higher searching efficiency and shorter handling time on maize-fed FAW indicate strong host-plant-mediated effects, which may be attributed to differences in larval quality, plant structural traits, and volatile cues that influence parasitoid host location and attack efficiency [89]. Such plant-driven variation in functional response parameters has been widely documented and is known to shape parasitoid efficacy in different crop systems [70]. Model selection further showed that Holling’s disc equation best fit the castor data, whereas Rogers’ random attack model better described the maize assays, reflecting differences in host depletion dynamics [65]. From an applied perspective, the results underscore the suitability of *B. hebetor* for deployment in maize-based agroecosystems. Enhanced fecundity, reduced developmental time, and increased searching efficiency suggest potential for field establishment and pest suppression, which remains to be validated under field conditions. The observed type II functional response further implies efficient control at lower host densities, although a tendency toward saturation at higher densities highlights the need for timely, early-season releases to maximize biocontrol efficacy [64,65].

Moreover, the future dynamics of pest populations and their NEs can shift considerably in response to variations in ambient temperature [90], food availability [91], moisture levels [92], and light conditions [93] substantially affecting their survival, development, and population buildup. In this investigation, projections of parasitoid populations and killing rates were higher on maize-fed larvae. This enhanced performance is supported by multiple indicators from our study, including shorter developmental durations, higher survival, increased fecundity and population parameters and greater killing efficiency. Similarly, host-plant-driven variation in prey quality has been shown to markedly influence population growth and predation projections of other NEs [94,95,96]. These results emphasize the significance of host-plant-mediated host suitability in shaping parasitoid success, providing critical insights for optimizing *B. hebetor* use in FAW biological control programmes.

## 5. Conclusions

This study indicates that host-plant-mediated trophic pathways substantially shape the developmental biology and parasitism performance of *B. hebetor*. Parasitoids emerging from the maize-fed FAW consistently exhibited accelerated development, higher survivorship, prolonged adult longevity, elevated fecundity, and superior population growth metrics compared with castor-fed FAW. Notably, effective parasitism and killing rate were markedly higher on maize-fed hosts, indicating overall more efficient host exploitation. Greenhouse experiments further confirmed these effects, revealing significantly higher effective parasitism rates and killing rates on maize-fed larvae. Functional response analyses further revealed that maize-fed hosts enhanced parasitoid foraging efficiency—reflected by greater searching ability and reduced handling time. These patterns underscore the pivotal role of host plant identity in determining parasitoid fitness and host exploitation capacity. Moving forward, detailed profiling of plant-derived nutritional and biochemical drivers, multi-generational assessments, and validation under field conditions are essential to unravel the mechanistic basis of these interactions. Integrating such insights into IPM frameworks will refine the deployment of *B. hebetor*, strengthen FAW suppression, and contribute to more resilient and sustainable crop protection systems.

## Figures and Tables

**Table 1:** Life history traits of *Bracon hebetor* parasitizing on *Spodoptera frugiperda* on castor and maize.

Parameters	Developmental Time (Day) ± SE				
Developmental time (day)	Developmental Stages	*n*	FAW-Castor	*n*	FAW-Maize
	Egg	100	2.85 ± 0.04 a	100	2.28 ± 0.05 b
	Larva	68	3.94 ± 0.03 a	75	3.35 ± 0.06 b
	Pupa	52	7.06 ± 0.11 a	62	6.44 ± 0.09 b
	Preadult	52	14.06 ± 0.11 a	62	12.18 ± 0.13 b
Adult longevity (day)	All	48	29.35 ± 0.37 b	55	31.11 ± 0.36 a
	Male	20	26.45 ± 0.23 b	22	28.04 ± 0.19 a
	Female	28	31.42 ± 0.09 b	33	33.15 ± 0.18 a
Total longevity (day)	Male	20	39.81 ± 0.28 b	22	40.70 ± 0.22 a
	Female	28	45.35 ± 0.21 a	33	45.60 ± 0.14 a

Standard errors were estimated using bootstrap resampling with 100,000 iterations. Pairwise differences among treatments were evaluated using a paired bootstrap test (*p* < 0.05). Means within a column followed by the same letter do not differ significantly.

**Table 2:** Reproductive parameters of *Bracon hebetor* parasitizing on *Spodoptera frugiperda* on castor and maize.

Parameters	FAW-Castor	FAW-Maize
APOP (Adult pre-oviposition period) (day)	0.678 ± 0.09 a	0.575 ± 0.08 a
TPOP (Total pre-oviposition period) (day)	14.60 ± 0.11 a	13.03 ± 0.23 b
Oviposition days	30.75 ± 0.14 b	32.58 ± 0.22 a
Fecundity (eggs/female)	226.50 ± 0.86 b	247.09 ± 0.41 a

Standard errors were estimated using bootstrap resampling with 100,000 iterations. Pairwise differences among treatments were evaluated using a paired bootstrap test (*p* < 0.05). Means within a column followed by the same letter do not differ significantly.

**Table 3:** Population parameters of *Bracon hebetor* parasitizing on *Spodoptera frugiperda* on castor and maize.

Parameters	FAW-Castor	FAW-Maize
Intrinsic rate of increase (*r*) (day^−1^)	0.186 ± 0.008 b	0.217 ± 0.008 a
Finite rate of increase (*λ*) (day^−1^)	1.205 ± 0.010 b	1.242 ± 0.011 a
Net reproductive rate (*R*_0_) (offspring/individual)	63.42 ± 10.182 a	81.54 ± 11.551 a
Gross reproductive rate (*GRR*) (offspring/individual)	145.05 ± 15.172 a	160.85 ± 15.129 a
Mean generation time (*T*) (day)	22.241 ± 0.205 a	20.263 ± 0.289 b
Doubling time (*DT*) (day)	3.714 ± 0.178 a	3.191 ± 0.133 b

Standard errors were estimated using bootstrap resampling with 100,000 iterations. Pairwise differences among treatments were evaluated using a paired bootstrap test (*p* < 0.05). Means within a column followed by the same letter do not differ significantly.

**Table 4:** Effective parasitism rates, non-effective parasitism rates and killing rates of *B. hebetor* parasitizing on *Spodoptera frugiperda* on castor and maize.

Parameters	FAW–Castor	FAW–Maize
Total effective parasitism rates of female	36.714 ± 0.793 b	46.393 ± 0.989 a
Net effective parasitism rate, *C*_0_	10.28 ± 1.665 b	15.31 ± 2.192 a
Stable effective parasitism rate, *ψ*	0.1594 ± 0.005 a	0.1679 ± 0.007 a
Finite effective parasitism rate, *ω*	0.1920 ± 0.006 a	0.2085 ± 0.009 a
Total non-effective parasitism rates of female	46.071 ± 1.906 a	49.242 ± 1.999 a
Net non-effective parasitism rate, *N*_0_	12.90 ± 1.086 a	16.25 ± 1.324 a
Finite non-effective parasitism rate, *ε*	0.1847 ± 0.009 b	0.2204 ± 0.006 a
Stable non-effective parasitism rate, *γ*	0.2295 ± 0.012 b	0.2656 ± 0.008 a
Total killing rate of females	82.785 ± 1.132 b	95.636 ± 0.832 a
Net killing rate, *Z*_0_	23.18 ± 3.735 b	31.56 ± 4.478 a
Finite killing rate,*θ*	0.3525 ± 0.008 b	0.3797 ± 0.003 a
Stable killing rate, *υ*	0.4381 ± 0.011 a	0.4576 ± 0.006 a
Transformation rate, *Q_p_*	1.365 ± 0.009 b	1.387 ± 0.006 a

Standard errors were estimated using bootstrap resampling with 100,000 iterations. Pairwise differences among treatments were evaluated using a paired bootstrap test (*p* < 0.05). Means within a column followed by the same letter do not differ significantly.

**Table 5:** Logistic regression estimates and standard errors (SE) for linear and quadratic coefficients describing the proportion of *Spodoptera frugiperda* larvae parasitized by *Bracon hebetor* on two host plant systems.

Host-Plant	Coefficient	Estimate	SE	Z Value	Pr (>|z|)	Type of Functional Response
Maize	Intercept (P_0_)	2.2430654	0.5064060	4.429	9.45 × 10^−6^ ***	Type II
	Linear (P_1_)	−0.1083065	0.0308329	−3.513	0.00044 ***	
	Quadratic (P_2_)	0.0007891	0.0003799	2.077	0.037800 *	
Castor	Intercept (P_0_)	0.6773491	0.4069013	1.665	0.0960 **.**	Type II
	Linear (P_1_)	−0.0699483	0.0277069	−2.525	0.0116 **	
	Quadratic (P_2_)	0.0004963	0.0003587	1.384	0.1665	

Significance codes: ‘***’ 0.001; ‘**’ 0.01; ‘*’ 0.05; ‘**.**’ 0.1; ‘ ’ 1.

**Table 6:** Functional response parameters—searching efficiency (a) and handling time (T_h_)—of *Bracon hebetor* on *Spodoptera frugiperda* estimated using Holling’s disc model and Roger’s random attack equation across two host plant systems.

Host-Plant	Models	Searching Efficiency (a) (h^−1^)	Handling Time (T_h_) in Days	Max. Parasitization Per Day (T/T_h_)	AIC
Maize	Holling’s disc equation	1.1252 ± 0.001	0.0556 ± 0.000	17.985	52.30
	Rogers’s random attack equation	3.3243 ± 0.872	0.0718 ± 0.008	13.927	51.32 *
Castor	Holling’s disc equation	0.7869 ± 0.142	0.0817 ± 0.014	12.239	56.57 *
	Rogers’s random attack equation	1.2464 ± 0.346	0.0912 ± 0.015	10.964	57.27

* Indication of best fit.

## Data Availability

The data that support the findings of this study are available from the corresponding author upon reasonable request.
